# Saliva microbiome, dietary, and genetic markers are associated with suicidal ideation in university students

**DOI:** 10.1038/s41598-022-18020-2

**Published:** 2022-08-22

**Authors:** Angelica P. Ahrens, Diego E. Sanchez-Padilla, Jennifer C. Drew, Monika W. Oli, Luiz F. W. Roesch, Eric W. Triplett

**Affiliations:** Microbiology and Cell Science Department, Institute of Food and Agricultural Sciences, University of Florida, Gainesville, FL USA

**Keywords:** Microbial communities, Microbiome, Microbiology, Neuroscience, Diseases of the nervous system, Depression

## Abstract

Here, salivary microbiota and major histocompatibility complex (MHC) human leukocyte antigen (HLA) alleles were compared between 47 (12.6%) young adults with recent suicidal ideation (SI) and 325 (87.4%) controls without recent SI. Several bacterial taxa were correlated with SI after controlling for sleep issues, diet, and genetics. Four MHC class II alleles were protective for SI including DRB1*04, which was absent in every subject with SI while present in 21.7% of controls. Increased incidence of SI was observed with four other MHC class II alleles and two MHC class I alleles. Associations between these HLA alleles and salivary bacteria were also identified. Furthermore, rs10437629, previously associated with attempted suicide, was correlated here with SI and the absence of *Alloprevotella rava*, a producer of an organic acid known to promote brain energy homeostasis. Hence, microbial-genetic associations may be important players in the diathesis-stress model for suicidal behaviors.

## Introduction

Suicide is the second leading cause of death in individuals between the ages of 10–34 in the United States^1^. Worldwide life-time prevalence of suicidal ideation (SI) was estimated to be 9.2% in a 2008 report of suicidal behaviors in 84,850 adults across 17 countries^2^. In a study of 67,000 degree-seeking college students across 108 institutions, 16,337 (24.3%) reported they harbored SI and 9.3% had made a suicide attempt^3^. The strongest association with suicidality, self-injury, suicide attempts, and mental health diagnosis in students was exposure to stress, in a dose-dependent manner^2^. Three or more exposures to stress resulted in 4.25 to 10.06 times higher endorsement of SI^3^, consistent with the diathesis-stress model of depression^4^. In 729 students, surveyed from 2002 to 2005, 11.1% endorsed having SI within the past 4 weeks, the majority of whom indicated they were not receiving treatment, and 16.5% reported at least one suicide attempt in their lifetime^4^. According to a 2020 CDC report, as many as one in four people in the U.S. between ages 18 and 24 had seriously contemplated suicide in the month prior^5^. Here, genetics, lifestyle (diet and sleep) factors, and salivary bacteria were explored for their associations with SI in 489 college students.

The genetic basis of SI risk is understudied, particularly in general population cohorts. Limited in number, genome-wide association studies (GWAS) and polygenic risk score studies have identified some possible genetic variants increasing in subjects with previous suicide attempts^6–9^, SI^7,9–11^, and other suicidal behavior^11^. However, this is often studied in restricted cohorts of individuals with schizophrenia^12^, bipolar disorder (BD)^6,11^, or other mood disorder^7,8^. Reports of suicide attempts in BD have yielded suggestive associations or gene variants with small effects^11^ or without definitive evidence of association^8^. Recent validation was attempted in studies of schizophrenia^6^ and BD, uncovering evidence for a role by cholesterol biosynthesis^13^. However, validation of other SNPs remains to be seen^13^. In one report, polygenic scores for major depressive disorder (MDD), but not for previous attempts of suicide, were predictive of SI^7^.

Evidence of autoimmunity or immune dysfunction in neurobiological and neuropsychiatric disorders^14^ warrant investigation into the mechanism by which immune haplotypes may play a role. Autoimmunity or immune dysfunction in neurobiological and neuropsychiatric disorders has been shown for schizophrenia^14^ and autism^15–19^ and human leukocyte antigen (HLA) genetics may play a moderate effect in depression^20^, although this connection is controversial in the literature. Furthermore, HLA alleles, particularly those that confer risk for type 1 diabetes, have been shown to influence the gut microbiome. However, the role of HLA in saliva microbiome composition has not been explored. Host immune surveillance systems recognize molecular patterns of microbiota, leading to inflammatory mediator recruitment. The role of HLA in depression, as well as in the saliva microbiome, remains to be elucidated.

Dietary influences on depression have been extensively described, with increasing attention on suicidal behaviors, and diet is also a confound of microbiome composition. Thus, it was of interest to the present investigation. Much of the nutritional psychiatry work has been performed in preclinical animal models and more human study is needed^21^. Clinical trials have suggested that the Mediterranean diet may prevent depression in patients with type 2 diabetes^22^, although the MoodFood trial demonstrated no benefit from a behavioral activation intervention and a reduction in MDD incidence was not observed after one year in overweight or obese individuals with subsyndromal depression^23^. Recently, a connection between fast food consumption and suicide attempts was reported in a cross-sectional study of adolescents from 32 countries, when controlling for food insecurity, obesity, physical exercise, and consumption of fruits and vegetables^24^.

While the literature on the gut-brain axis is growing, the oral microbiome has garnered little attention. Microbe-brain interactions play a consequential role in governing peripheral and central nervous system events such as cytokine production, short chain fatty acid release, microglial maturation and activation^25^, as well as expression of the synaptic plasticity-related gene, brain-derived neurotrophic factor (BDNF) in the amygdala and hippocampus^26^. Weakening of intestinal and mucosal layers facilitates communication of microbiota to the brain and can cause chronic, low-grade inflammation, as seen in depression^27^. The oral-gut-brain axis describes a pathway by which oral microbiota may influence neurological and systemic disorders either directly or indirectly, including periodontitis and non-oral systemic disease^28^. Within the saliva resides a complex network of microbes that closely represents the structure of commensal bacteria in the gut^29^. Microorganisms in the oral cavity are important for host health, as they deter exogenous organisms, contribute to pH dynamics required for a healthy mouth, and can influence nutrient supply in the oral cavity^30^. These microorganisms are found on both hard and soft oral tissues, with an estimated 100–200 phylotypes in every individual^31^. Saliva plays several important roles^32^ including: (1) delivery of innate and adaptive defense to the host, (2) provision of nutrients that support the growth of pathogenic or health-promoting microorganisms, (3) removal of substrates, and (4) regulation of pH within the oral cavity. It is the interaction with host that determines whether an oral bacterium is commensal or pathogenic^31^. Periodontal pathogens can insert into the periodontal pocket epithelium, where they can then enter the bloodstream and promote infection in the body and brain. Species such as *Porphyromonas gingivalis*, *Tannerella forsythia*, and *Treponema denticola* have been associated with periodontitis. Both gingivitis and periodontitis^33^, a chronic inflammatory condition, have been linked to cardiovascular health, diabetes, depression, gastrointestinal disease, Alzheimer’s, and certain cancers, suggesting a two-way effect between oral and systemic disease^28^. Emerging literature suggests that the oral microbiome may be leveraged for treatment and diagnosis of oral as well as systemic disease. Oral disease prevention strategies have been proposed to reduce burden.

Our understanding of the role of oral bacteria in the systemic pathophysiology of depression is limited and, to our knowledge, links with SI have not yet been explored in human populations. Saliva is an understudied entryway of bacteria into the human body and is easily sampled. In this investigation, saliva microbiome, genetics, and dietary habits were studied to identify risk indicators or factors associated with increased incidence of recent SI in a cohort of 489 college students. We sought to examine whether bacterial genera, species, and high-resolution amplicon sequence variants (ASVs) are associated with recent SI were identified, even when controlling for sex, dietary habits, and sleep. Bacterial associations were assessed by two approaches: across the general cohort as well as by accounting for lifestyle (diet and sleep) confounds. As diet is a major contributor to saliva microbiome composition, an assessment of diet was required to understand the interplay of the microbiome with host genetics and SI^34^. In particular, microbiome composition was examined in the context of reported sleep issues given that chronic fatigue has been linked to dysbiosis^35^ and insomnia is considered a risk factor for SI, suicidal behavior, and suicide^36,37^. Fourteen SNPs with published evidence of association to suicidal behaviors were assessed for links to SI and subsequently, salivary microbiota. HLA connections with SI and salivary microbes were also examined.

## Results

### Description of the cohort and survey instruments used

Mediterranean Diet Quality Index (KIDMED) and Patient Health Questionnaire-9 (PHQ-9) responses are provided in Table 1. The cohort was comprised of 489 total students of whom 318 were female (65.0%) and 171 were male; the average age was 21.37 ± 4.14 years. Of the 489 subjects, 60 (12.3%) screened positive for SI, as defined by a score ≥ 1 on question 9 of the PHQ-9, and 109 (22.3%) scored ≥ 10 on the PHQ-9, which is the suggested cut-off for Major Depressive Disorder (MDD) with optimal sensitivity and specificity according to Kroenke et al.^38^. Of the 109 subjects (22.3%) who scored ≥ 10 on the PHQ-9, 64 scored within the moderate, 33 within moderately severe, and 12 within severe ranges, respectively. A total of 166 subjects (34.0%) scored in the mild range for depression (PHQ-9 score of 5–9), while 214 (43.8%) scored in the none to minimal depression range (PHQ-9 score of 0–4). The KIDMED questions, as presented to the participants in the electronic survey, are provided in Supplementary Table 1.

**Table 1:** Diet and depression distributions in the cohort.

	N	KIDMED (mean ± SD)	95% CI
**Depression level**			
Suicidal ideation (SI)	59	4.46 ± 2.44	0.64
Depression (moderate to severe)	60	4.58 ± 2.66	0.69
Mild depression	155	4.42 ± 2.77	0.44
None or minimal depression	213	5.32 ± 2.60	0.35
**PHQ-9 severity**			
Severe (PHQ-9 ≥ 20)	12	4.00 ± 2.37	1.51
Moderately severe (PHQ-9 15–19)	33	4.24 ± 2.92	1.03
Moderate (PHQ-9 10–14)	63	4/71 ± 2.47	0.62
Mild (PHQ-9 5–9)	165	4.45 ± 2.73	0.42
None to minimal (PHQ-9 0–4)	214	5.31 ± 2.60	0.35

Distribution of Patient Health Questionnaire-9 PHQ-9 and KIDMED total scores across 489 subjects: Depression level categories defined as follows: screened positive for recent suicidal ideation (SI) based on a score of ≥ 1 on question 9 of the PHQ-9 (SI), regardless of PHQ-9 score; did not endorse recent SI and had a PHQ-9 total score of 0–4 (None or minimal depression), 5–9 (Mild depression), or ≥ 10 (Depression, moderate to severe). Distribution of PHQ-9 scores is then provided based on the stratification by Kroenke et al.: PHQ-9 total scores, 0–4 (None to minimal), 5–9 (Mild), 10–14 (Moderate), 15–19 (Moderately severe), ≥ 20 (Severe). Two subjects (one with SI and moderate depression and one with NOSI and mild depression) are not presented as their KIDMED had missing values, thus the KIDMED index could not be calculated.

The KIDMED and PHQ-9 total scores followed a non-normal distribution (female N = 317 and male N = 170; all *p* values < 0.026) and had a low, negative correlation (Spearman ρ = − 0.150, N = 487). PHQ-9 scores were statistically higher in females ($\overline{x}$ = 7.0 ± 5.4, $\tilde{x}$ = 6.0) than males ($\overline{x}$ = 5.3 ± 4.6, $\tilde{x}$ = 4.0, *p* = 0.001), although total KIDMED scores did not differ significantly (*p* = 0.082). Females were more likely to report having had SI in the past 2 weeks, χ^2^ (1, *N* = 489) = 5.3, *p* = 0.021, comprising 78.3% of those with SI but only 65% of the cohort.

Total KIDMED scores did not differ significantly between individuals with or without SI, nor across traditional PHQ-9 severity levels, excepting a higher score in those with “no” depression ($\overline{x}$ = 5.3 ± 2.6, $\tilde{x}$ = 5.0) compared to those with “mild” ($\overline{x}$ = 4.4 ± 2.7, $\tilde{x}$ = 4.0, *p* < 0.001).

### Incidence of SI increases with the frequency of reported sleep issues

Overall, individuals with SI (n = 59) were more likely to endorse all eight of the other symptoms captured in the PHQ-9 than individuals without SI (n = 428, *p* < 0.001 for each comparison). Specifically, females who reported having issues with sleep nearly every day in the past 2 weeks were significantly more likely to endorse SI (OR = 23.5 [6.6–83.9], *p* < 0.0001). A higher proportion of females (66.0%) with SI and males with SI (38.5%) had diminished sleep most days within the past 2 weeks, compared to females (28.0%) and males (24.1%) without SI, respectively.

### Regular consumption of nuts is inversely correlated with incidence of SI

Individuals who reported consuming nuts less than 2–3× weekly (*N* = 489, χ^2^ = 10.5, *p* = 0.001) were also more likely to endorse recent SI. In addition, females who reported not consuming a second fruit or fruit juice daily (*N* = 317) χ^2^ = 3.2, *p* = 0.072), trended toward being more likely to express SI in the past 2 weeks. Fewer females with SI (14.6%) reported consuming at least two fruits a day, compared to 24.7% of those without (24.7%). No single dietary habit on the KIDMED correlated with SI in males, possibly attributable to the low incidence of SI in males.

### HLA Class II DQA1*01, DRB1*15, DRB3*02, DPB1*01/*05 and HLA Class I C*08 and A*30 are positively associated with incidence of SI, while DRB1*04, DQA1*03, DQB1*03, DRB3*01, and DQ8 appear protective

Five HLA haplotypes appear to be protective of SI, while eight were significantly associated with increased risk (see Table 2). Full statistics for all chi square analyses performed across the two HLA class I and eight HLA class II genes are provided in Supplementary Table 2.

**Table 2:** Human Leukocyte Antigen (HLA) haplotypes associated with incidence of suicidal ideation (SI).

	No SI (n = 254)		SI (n = 35)		Risk	Protective	OR	95% CI	χ^2^	*p*
	Present	Absent (%)	Present	Absent (%)						
DRB1*04	63	191 (75.2)	0	35 (100.0)		x	0.0425	0.0026–0.7025	–	0.0001
DRB1*15	64	190 (74.8)	14	21 (60.0)	x		1.9792	0.9507–4.1201	3.4	0.065

	No SI (n = 268)		SI (n = 34)							
	Present	Absent (%)	Present	Absent (%)						
DQA1*01	175	93 (34.7)	27	7 (20.6)	x		2.0498	0.8600–4.8855	2.7	0.0995
DQA1*03	84	184 (68.7)	2	32 (94.1)		x	0.1369	0.0321–0.5846	–	0.001

	No SI (n = 258)		SI (n = 34)							
	Present	Absent (%)	Present	Absent (%)						
DQB1*03	150	108 (41.9)	13	21 (61.8)		x	0.4457	0.2138–0.9291	4.8	0.028

	No SI (n = 272)		SI (n = 35)							
	Present	Absent (%)	Present	Absent (%)						
DRB3*01	65	207 (76.1)	2	33 (94.3)		x	0.193	0.0451–0.8263	–	0.015
DRB3*02	94	178 (65.4)	18	17 (48.6)	x		2.005	0.9873–4.0717	3.8	0.051

	No SI (n = 260)		SI (n = 34)							
	Present	Absent (%)	Present	Absent (%)						
DPB1*01	35	225 (86.5)	8	26 (76.5)	x		1.978	0.8297–4.7156	2.4	0.118
DPB1*05	22	238 (91.5)	7	27 (79.4)	x		2.8047	1.0966–7.1733	5.0	0.026

	No SI (n = 264)		SI (n = 35)							
	Present	Absent (%)	Present	Absent (%)						
C*08	27	237 (89.8)	7	28 (80.0)	x		2.1944	0.8754–5.5008	2.9	0.087

	No SI (n = 267)		SI (n = 34)							
	Present	Absent (%)	Present	Absent (%)						
A*30	17	250 (93.6)	6	28 (82.4)	x		3.1513	1.1485–8.6466	5.4	0.0197

	No SI (n = 252)		SI (n = 33)							
	Present	Absent (%)	Present	Absent (%)						
DQ2.2	52	200 (79.4)	11	22 (66.7)	x		1.9231	0.8768–4.2181	2.7	0.098
DQ2.5	49	203 (80.6)	7	26 (78.8)			N.S.		N.S.	N.S.
DQ2.2 and DQ2.5	13	239 (94.8)	5	28 (84.8)	x		3.283	1.0893–9.8944	4.9	0.026
DQ8	72	180 (71.4)	2	31 (93.9)		x	0.1613	0.0376–0.6916		0.0051

χ^2^ and odds ratio (OR) results for HLA haplotypes that were near or significant. Fisher exact test was computed where χ^2^ is not listed. Risk indicators associate with increased incidence of SI, while protective factors associate with decreased incidence.

Compared to those with NOSI, subjects with SI were more likely to have DQA1*01 homozygosity than to not have any DQA1*01 alleles, χ^2^ (1, *N* = 302) = 6.3, *p* = 0.012, and subjects who had at *least* one DQA1*01 allele were slightly more likely to have screened positive for SI, χ^2^ (1, *N* = 302) = 2.7, *p* = 0.0995. Subjects who screened positive for SI were twice as likely to carry DRB3*02, χ^2^ (1, *N* = 307) = 3.8, *p* = 0.051, and 1.98 times more likely to carry at least one allele of DRB1*15, χ^2^ (1, *N* = 289) = 3.4, *p* = 0.065. Furthermore, subjects with SI were more likely to be homozygous for DRB1*15, χ^2^ (1, *N* = 222) = 7.3, *p* = 0.0069. Subjects with SI were two to three times more likely to carry DPB1*01 or DPB1*05, respectively. Alleles C*08 and A*30 were also observed at higher frequencies in individuals with SI. Those with SI were also more likely to be DQ2.2 and DQ2.5, χ^2^ (1, *N* = 285) = 4.9, *p* = 0.026. 15.2% of SI were DQ2.2 and DQ2.5 compared to only 5.2% of subjects with no SI.

Regarding the protective haplotypes, subjects with DQA1*03 were 7.3× more likely to not have SI, *p* = 0.001. Subjects without DQB1*03 were 2.2× more likely to screen negative for SI, χ^2^ (1, *N* = 292) = 4.8, *p* = 0.028, than subjects with DQB1*03. Subjects who had at *least* one DRB1*15 allele were slightly more likely to have screened positive for SI, χ^2^ (1, *N* = 289) = 3.4, *p* = 0.064. No individual with SI was homozygous for DQ8 χ^2^ (1, *N* = 289) = 6.2, *p* = 0.0051, or had DR4–DQ8, χ^2^ (1, *N* = 270) = 8.8, *p* = 0.003, the latter, a haplotype which was observed in 21.5% of subjects with no SI. Only 6.1% of subjects with SI had DQ8 compared to 28.6% of those with no SI; those with DQ8 were 6.2× more likely to not endorse SI, χ^2^ (1, *N* = 285) = 6.2, *p* = 0.0051. Those with DRB1*04 (*p* = 0.0001) and DQ8 were 23.5 and 6.2 times less likely to report recent SI, respectively. Subjects with DRB3*01 were 5.2× more likely to not have SI (*p* = 0.015).

### Presence of minor G allele at rs10437629 is associated with 2.6× increased incidence of SI

**Figure 1:**
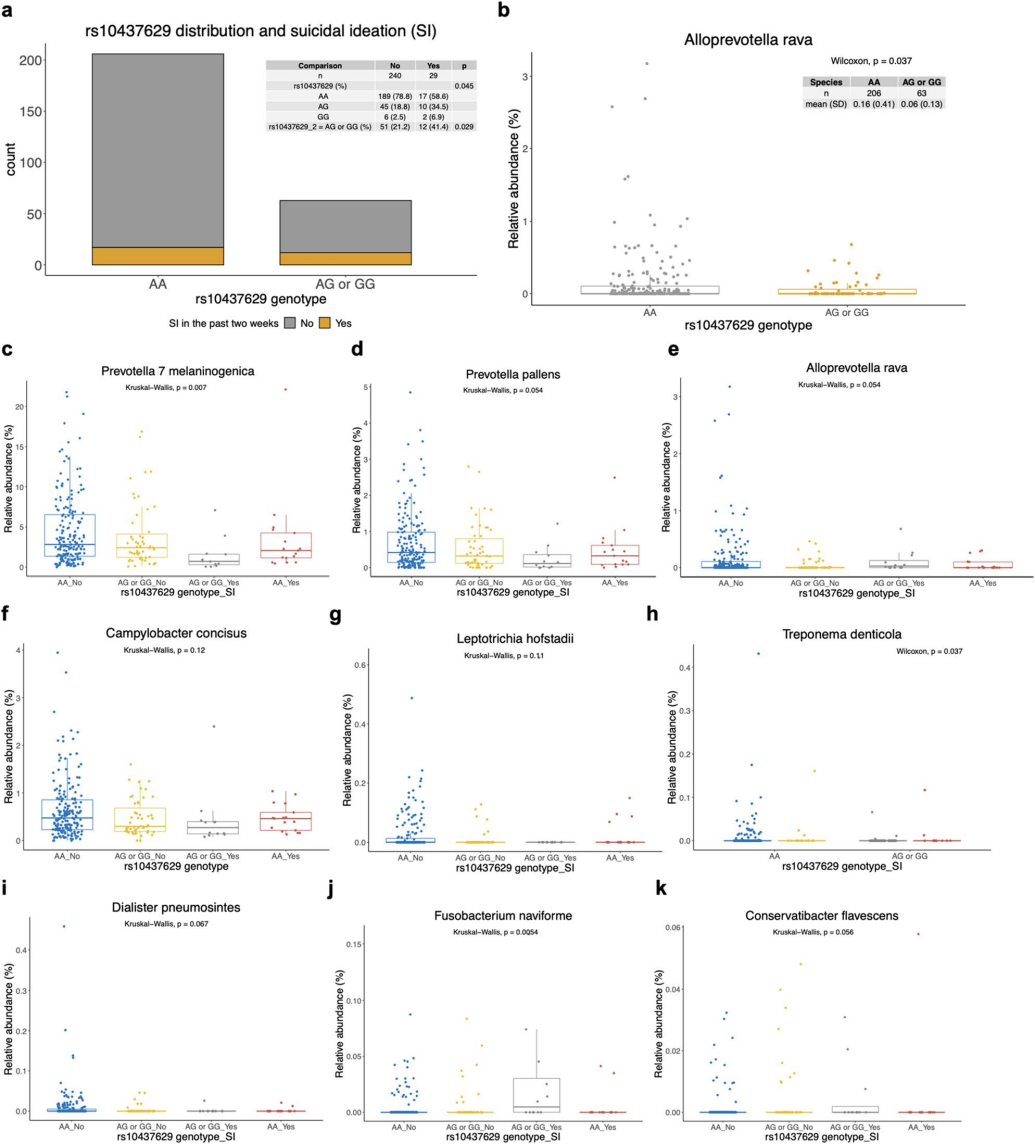
Interaction of rs10437629 genotype and endorsement of recent suicidal ideation (SI) with significantly associated saliva species. (**a**) Distribution of A and G alleles in rs10437629 (N = 269). (**b**) Relative abundance of *Alloprevotella rava* counts across genetic variants and SI, with a significant difference in abundances between individuals with AA and individuals with AG or GG, Wilcoxon *p* = 0.037. Here, two groups were compared (homozygotes AA versus heterozygotes, AG, or homozygotes, GG, for the minor G allele) with a Wilcoxon test. (**c**–**k**) Selected bacteria that were differentially abundant across rs10437629 genotype (AA vs. AG or GG) and endorsement of SI. For these associations, Kruskal–Wallis tests were performed against four separate genotype-SI combinations: (1) AA genotype and no SI (AA_No), (2) AG or GG genotype and no SI (AA or GG_No), (3) AG or GG and SI (AG or GG_Yes), and (4) AA genotype and SI (AA_Yes). In e, *Alloprevotella rava* is again presented but with this four-group comparison. For each four-group comparison, a Kruskal Wallis test was conducted; *p* values are presented within the plots. Species assignment was based on Silva taxonomy of the amplicon sequence variants (ASVs) and included all ASVs that were mapped to that closest cultured relative.

Recent SI in the cohort was assessed with respect to genotype at available single nucleotide polymorphisms (SNP) with a prior connection to suicidal attempts, suicide, or suicidality per EBI annotation in the NHGRI-EBI GWAS catalog. Of the sixteen SNPs acquired by the PMRA, fourteen were observed at a minor allele frequency (MAF) of ≥ 0.10 in our cohort and were subsequently tested against incidence of SI. rs10437629, which has been associated with attempted suicide in bipolar disorder^6^, was the only SNP found to be significantly associated with SI, with the minor G allele conferring increased incidence of SI (OR = 2.62 [1.17 to 5.828, 95% CI], *p* = 0.0186) at a MAF of 0.132. Data for this SNP was available for 269 individuals (Fig. 1). The increased incidence of SI was observed in both heterozygotes and homozygotes, although the presence of only one G allele was sufficient and homozygosity for G was rare (Fig. 1). While 8.3% of individuals homozygous for the A allele expressed SI, the incidence was 19.0% for those possessing at least one G allele at rs10437629.

Differences in the saliva microbiota were also observed with respect to the interaction of this genotype with recent SI (Fig. 1). *Alloprevotella rava* was rarely seen in individuals with AG or GG (Fig. 1b, *p* = 0.037 for two group comparison), and more specifically was highest in abundance in those with AA and without SI (Fig. 1e, *p* = 0.054 across the four groups). *Prevotella melaninogenica*, which was a significant microbe present as high as 20% relative abundance in individuals AA and without SI, had the lowest abundances in individuals with SI and possessing the G allele (Fig. 1c, *p* = 0.007). *Prevotella pallens* was highest in those with AA and without SI (*p* = 0.054). *Campylobacter concisus* was highest in individuals with AA, whether they expressed recent SI or not, but was lowest in those with the G allele and expressing SI (Fig. 1f, *p* = 0.12).

Although observed at low relative abundances and prevalence, significant differences were observed in the abundance of *Treponema denticola* (Fig. 1h, *p* = 0.037) and *Fusobacterium naviforme* (Fig. 1j, *p* = 0.0054), and near significant differences were observed across the four groups with respect to *Leptotrichia hofstadii* (Fig. 1g, *p* = 0.11), *Dialister pneumosintes* (Fig. 1i, *p* = 0.067), and *Conservatibacter flavescens* (Fig. 1k, *p* = 0.056), which were all seen at the highest abundance in individuals who were AA and did not have SI. No individual who had the G allele and expressed recent SI had *Leptotrichia hofstadii* in their saliva, and *Dialister pneumosintes* was only observed in one of these subjects.

### Dietary confounders of saliva microbiome diversity

Saliva microbiome data was available for a total of 372 individuals. Microbial divergence of non-SI controls (N = 325) and individuals with SI (N = 47) was observed at the community-level by the Bray–Curtis dissimilarity measure (*p* = 0.047). Dissimilarity appears to be regulated in part by sex and regular pasta or rice consumption (Supplementary Table 1, *p*’s < 0.05). Variance is also explained by daily consumption of fruit and whether the subject visits a fast-food hamburger restaurant more than once weekly (*p*’s < 0.1), but not by overall KIDMED compliance score (*p* = 0.697).

### Microbial diversity indices are lower in individuals with SI in the presence of sleep issues

Microbiome diversity differences in suicidal ideation (SI) endorsement concurrent with the presence of sleep issues were assessed. Shannon and Diversity Coverage indices (*p*’s = 0.045 and 0.036, Supplementary Fig. 1b,d) were significantly lower in SI_sleep_. Diversity Gini Simpson, Inverse Simpson, and Evenness Simpson were lower in individuals with SI, although at *p* ≤ 0.1. Richness in NOSI_sleep_ and SI_sleep_ was not significantly different (Supplementary Fig. 1g,h).

### Network analysis of salivary microbiota observed in individuals with sleep issues

Bacterial networks of salivary genera were assessed in the saliva of individuals with sleep issues with correlation analysis (Supplementary Fig. 2). Spearman correlation coefficients from the network analysis are provided in Supplementary Table 3.

Overall, *Veillonella* had strong negative correlations with genera including *Actinomyces*, *Alloprevotella*, *Bergeyella*, *Campylobacter*, *Granulicatella*, *Oribacterium*, *Prevotella*, *Rothia*, and *Stomatobaculum* (Spearman correlations < − 0.4; Supplementary Fig. 2a) and was positively correlated with *Megasphaera*, *Saccharimonadaceae TM7x*, and *Selenomonas* (Spearman correlations > 0.45; Supplementary Fig. 2a), all three of which were more abundant in individuals with SI (Supplementary Fig. 2b). Genera that negatively correlated with Veillonella were observed at higher abundance in controls (Supplementary Fig. 2b). *Granulicatella* correlated positively with *Streptococcus, Rothia, Haemophilus, Porphyromonas, Fusobacterium, Gemella, Actinomyces,* and *Aggregatibacter* (Spearman correlation between 0.649 and 0.362, Supplementary Fig. 2c) and negatively with *Selenomonas*, while *Alloprevotella* correlated positively with *Neisseria*, *Haemophilus*, *Eubacterium nodatum group*, and *Peptostreptococcus* (Supplementary Fig. 2d). *Prevotella* and *Atopobium* (higher in NOSI_sleep_), and *Selenomonas*, *Veillonella*, and *Saccharimonadaceae TM7x* (higher in SI_sleep_) were the top five genera most positively correlated with *Megasphaera* (Supplementary Fig. 2e). *Haemophilus*, *Porphyromonas*, *Capnocytophaga*, *Gemella*, and *Fusobacterium* were higher in NOSI_sleep_ and were the top five genera most negatively correlated with *Megasphaera*.

### Differentially expressed ASVs in individuals with SI

Using DESeq2, normalized base mean counts of the ASVs observed in the saliva community were compared in four methods: (1) the full cohort: those with SI compared to those with no SI, NOSI, and then selected cohorts, where those with SI were compared to (2) those with PHQ-9 scores above 10 (the published threshold for MDD), DEP, (3) those with PHQ-9 scores < 5, NODEP, and (4) those with PHQ-9 scores between 5 and 9, MILD), separately. Depending on the pairwise group comparison, between 240 and 439 ASVs were found to be differentially abundant between those with SI and those without (Supplementary Fig. 3; see Supplementary Tables 4–7 for full statistics of all significant ASVs). SI and NOSI groups demonstrated the greatest number of significantly different ASVs, while SI and DEP had the fewest, suggesting that their microbial communities were more similar, although still distinctive.

The four models shared 75 differentially abundant ASVs in common (Supplementary Fig. 3e), of which 31 were more abundant in individuals with SI and 44 were more abundant in all four comparative groups without SI. Full statistics and taxonomy for these ASVs are provided in Table 3. *Bacteroides dorei* (ASV509), *Streptococcus* undetermined (ASV396), *Selenomonadaceae* undetermined (ASV333), and *Klebsiella oxytoca* (ASV163) were at least four times higher in individuals with SI, irrespective of the comparative groups’ total PHQ-9 scores (Table 2). Consistently elevated at least four-fold in the non-SI groups were *Alloprevotella tannerae* (ASV250), *Alloprevotella* undetermined (ASV115), *Prevotella 7 melaninogenica* (ASV147), *Veillonella parvula* (ASV180), *Leptotrichia* undetermined (ASV289 and ASV179), *Saccharimonadales* undetermined (ASV336), and *Klebsiella michiganensis* (ASV58), irrespective of PHQ-9 score (Table 3).

**Table 3:** Amplicon sequence variants (ASVs) elevated in students with suicidal ideation (SI) or without.

ASV ref	SILVA 138 taxonomic assignment	No SI				Mild depression				No or minimal depression				Depressed			
		BaseMean	log2FC	*p*	padj	BaseMean	log2FC	*p*	padj	BaseMean	log2FC	*p*	padj	BaseMean	log2FC	*p*	padj
**Elevated in individuals with SI**																	
ASV982	*Actinomyces*	1.98	0.72	0.0059	0.0254	1.99	0.91	0.0015	0.0149	1.65	1.28	2.71E−07	1.32E−05	2.18	1.25	0.0014	0.0113
ASV1924	*Cryptobacterium curtum*	1.43	1.09	3.69E−09	1.73E−07	1.65	1.08	5.64E−06	0.0002	1.58	1.10	5.49E−07	2.34E−05	2.00	1.08	0.0025	0.0173
ASV509	*Bacteroides dorei*	5.39	*4.02*	1.11E−47	1.79E−44	3.63	*2.38*	2.73E−14	9.09E−12	5.38	*3.65*	1.19E−32	3.19E−29	5.46	*2.48*	1.34E−07	6.07E−06
ASV451	*Prevotella_7 melaninogenica*	1.97	1.13	8.35E−06	0.0001	2.36	1.02	0.0008	0.0096	1.76	1.69	1.24E−11	1.66E−09	2.72	1.23	0.0033	0.0207
ASV914	*Prevotella oulorum*	1.34	0.96	2.93E−06	5.20E−05	1.57	0.86	0.0009	0.0101	1.44	1.00	2.27E−05	0.0005	1.70	1.20	0.0011	0.0095
ASV1765	*Prevotella oris*	1.32	0.81	7.60E−05	0.0007	1.42	0.87	0.0003	0.0042	1.48	0.71	0.0035	0.0316	1.59	1.06	0.0029	0.0192
ASV642	*Capnocytophaga gingivalis*	3.21	1.51	1.63E−07	4.25E−06	3.72	1.67	4.36E−07	2.37E−05	3.97	1.35	4.65E−05	0.0009	4.95	1.77	9.86E−05	0.0015
ASV396	*Streptococcus*	6.32	*3.41*	4.15E−25	2.24E−22	5.90	*2.56*	6.10E−12	1.42E−09	6.22	*2.07*	5.09E−08	3.18E−06	8.47	*3.89*	4.80E−14	1.13E−11
ASV1753	*Streptococcus gordonii*	1.33	1.14	1.76E−08	5.91E−07	1.69	0.89	0.0009	0.0100	1.37	1.33	6.35E−09	5.51E−07	1.82	1.33	0.0006	0.0059
ASV333	*Selenomonadaceae*	4.81	*2.86*	6.12E−20	2.47E−17	6.55	*3.48*	1.40E−22	2.56E−19	6.05	*3.29*	2.70E−22	2.42E−19	5.36	*2.99*	4.92E−10	5.77E−08
ASV121	*Veillonella*	49.93	*2.37*	9.59E−07	1.93E−05	49.72	1.49	0.0049	0.0394	48.46	1.43	0.0064	0.0452	24.75	1.66	0.0070	0.0370
ASV268	*Veillonella atypica*	4.15	1.80	7.92E−08	2.32E−06	5.01	1.97	2.44E−07	1.50E−05	3.97	*2.56*	1.61E−14	2.88E−12	6.33	*3.44*	2.04E−11	3.99E−09
ASV296	*Veillonella atypica*	2.12	1.57	2.46E−09	1.24E−07	2.99	1.18	0.0005	0.0066	1.94	*2.32*	2.30E−19	1.24E−16	3.15	*2.32*	2.82E−07	1.10E−05
ASV1187	*Selenomonadaceae*	1.59	1.57	4.25E−13	3.70E−11	2.01	1.49	7.77E−08	5.33E−06	1.86	1.56	2.45E−09	2.35E−07	2.48	1.90	4.98E−06	0.0001
ASV381	*Selenomonas sputige*	9.57	1.52	2.40E−05	0.0003	6.45	1.14	0.0016	0.0158	10.49	1.68	1.19E−05	0.0003	7.52	1.43	0.0026	0.0177
ASV836	*Veillonella dispar*	1.98	1.18	1.47E−06	2.79E−05	2.18	1.32	2.43E−06	8.73E−05	2.10	1.31	1.15E−06	4.23E−05	2.62	1.67	3.66E−05	0.0007
ASV612	*Veillonella*	3.58	1.07	0.0006	0.0042	3.53	1.46	9.73E−06	0.0003	3.97	1.06	0.0020	0.0204	4.70	1.34	0.0034	0.0207
ASV1248	*Veillonella dispar*	1.22	0.99	1.04E−07	2.96E−06	1.37	1.00	1.87E−05	0.0005	1.35	0.97	1.65E−05	0.0004	1.61	1.09	0.0023	0.0162
ASV2094	*Dialister*	1.46	0.87	8.57E−05	0.0008	1.62	0.87	0.0007	0.0081	1.34	1.25	1.47E−08	1.10E−06	1.75	1.25	0.0007	0.0072
ASV1261	*Veillonella*	1.64	0.69	0.0046	0.0208	1.53	1.06	4.37E−05	0.0009	1.35	1.28	2.22E−08	1.57E−06	1.78	1.29	0.0007	0.0072
ASV548	*Leptotrichia*	4.89	1.42	6.68E−06	9.90E−05	5.40	1.67	2.48E−06	8.76E−05	6.19	1.17	0.0009	0.0111	7.21	1.77	0.0003	0.0035
ASV650	*Fusobacterium nucleatum*	2.24	0.90	0.0009	0.0057	2.27	1.14	0.0001	0.0019	2.18	1.15	4.54E−05	0.0009	2.47	1.90	3.69E−06	9.62E−05
ASV243	*Saccharimonadales*	17.55	1.54	6.50E−05	0.0007	21.90	1.48	0.0005	0.0063	20.07	1.58	0.0002	0.0025	28.24	1.56	0.0059	0.0323
ASV592	*Saccharimonadaceae TM7x*	5.56	1.18	0.0003	0.0022	6.23	1.27	0.0004	0.0051	6.47	1.08	0.0023	0.0226	7.79	1.30	0.0054	0.0300
ASV1462	*Saccharimonadaceae TM7x*	1.58	0.88	0.0002	0.0014	1.57	1.16	5.28E−06	0.0002	1.66	0.94	0.0002	0.0032	1.86	1.38	0.0003	0.0039
ASV285	*Neisseria mucosa*	2.74	1.48	2.71E−07	6.54E−06	3.32	1.48	1.93E−05	0.0005	3.12	1.51	2.98E−06	9.31E−05	4.00	*2.09*	8.10E−06	0.0002
ASV1044	*Neisseria*	1.28	0.93	1.58E−06	2.94E−05	1.59	0.69	0.0065	0.0482	1.29	1.12	1.71E−07	9.04E−06	1.63	1.12	0.0016	0.0125
ASV855	*Neisseria*	1.28	0.83	8.73E−05	0.0008	1.30	1.03	2.91E−05	0.0006	1.26	1.03	8.56E−06	0.0002	1.57	1.05	0.0049	0.0274
ASV163	*Klebsiella oxytoca*	2.10	*2.67*	4.99E−29	4.03E−26	2.88	*2.99*	2.20E−22	2.56E−19	2.61	*3.00*	4.98E−26	6.70E−23	4.72	*2.98*	2.04E−09	1.71E−07
ASV1017	*Aggregatibacter*	1.56	0.92	6.04E−05	0.0006	1.59	1.14	7.04E−06	0.0002	1.68	0.94	0.0003	0.003754999	1.87	1.39	0.0003	0.0036
ASV733	*Haemophilus parainfluenzae*	1.69	0.88	0.0004	0.0026	1.79	0.98	0.0004	0.0054	1.56	1.25	4.09E−07	1.90E−05	1.95	1.47	0.0002	0.0029

ASV ref	SILVA 138 taxonomic assignment	No SI				Mild depression				No or minimal depression				Depressed			
		BaseMean	log2FC	*p*	padj	BaseMean	log2FC	*p*	padj	BaseMean	log2FC	*p*	padj	BaseMean	log2FC	*p*	padj
**Elevated in individuals without SI**																	
ASV802	*Actinomyces graevenitzii*	3.13	− 1.76	5.61E−07	1.22E−05	1.90	− 1.13	0.0004	0.0048	3.80	− 2.19	1.21E−08	9.31E−07	1.76	− 1.41	7.32E−05	0.0013
ASV294	*Actinomyces*	12.96	− 2.34	4.42E−09	1.93E−07	7.56	− 1.72	2.10E−05	0.000499624	11.62	− 2.32	1.09E−08	8.65E−07	11.94	− 3.01	2.54E−09	1.99E−07
ASV583	*Paludibacteraceae F0058*	2.86	− 1.02	0.0006	0.0040	2.46	− 0.89	0.0037	0.0320	2.88	− 1.12	0.0003	0.0046	2.17	− 1.00	0.0029	0.0189
ASV614	*Alloprevotella rava*	2.77	− 1.43	1.96E−05	0.0002	2.18	− 1.19	0.0003	0.0038	2.69	− 1.49	1.62E−05	0.0004	2.37	− 1.80	4.57E−06	0.0001
ASV581	*Tannerella*	4.53	− 1.50	7.47E−06	0.0001	3.95	− 1.44	2.22E−05	0.0005	4.13	− 1.47	2.66E−05	0.0006	3.64	− 1.78	1.16E−05	0.0003
ASV394	*Porphyromonas*	4.35	− 1.73	6.99E−07	1.45E−05	3.27	− 1.44	1.82E−05	0.0005	3.38	− 1.45	2.92E−05	0.0006	3.01	− 1.77	1.11E−05	0.0002
ASV681	*Alloprevotella tannerae*	3.06	− 1.76	6.69E−07	1.42E−05	2.63	− 1.70	1.82E−06	7.18E−05	1.64	− 0.88	0.0039	0.0331	1.56	− 1.15	0.0013	0.0109
ASV414	*Alloprevotella rava*	3.13	− 1.77	5.43E−07	1.20E−05	2.53	− 1.60	6.38E−06	0.0002	2.90	− 1.77	6.40E−07	2.61E−05	1.64	− 1.26	0.0004	0.0049
ASV674	*Prevotella_7*	3.25	− 1.82	3.06E−07	7.27E−06	1.85	− 1.09	0.0006	0.0072	2.57	− 1.58	5.82E−06	0.0002	2.39	− 2.01	4.27E−07	1.47E−05
ASV240	*Porphyromonas*	6.40	− 2.52	4.66E−10	2.59E−08	4.55	− 2.20	4.60E−08	3.25E−06	2.21	− 0.99	0.0023	0.0226	11.13	− 4.20	4.29E−17	5.03E−14
ASV250	*Alloprevotella tannerae*	15.48	− 3.22	1.05E−14	1.21E−12	18.00	− 3.67	3.36E−17	1.96E−14	7.92	− 2.37	4.22E−09	3.78E−07	8.26	− 3.09	4.36E−11	6.40E−09
ASV213	*Prevotella_7*	9.64	− 3.43	2.15E−15	2.89E−13	9.71	− 3.67	1.02E−16	4.74E−14	5.03	− 2.62	1.99E−10	2.23E−08	2.25	− 1.90	1.95E−06	5.46E−05
ASV115	*Alloprevotella*	34.77	− 3.64	1.12E−16	1.82E−14	27.94	− 3.55	6.80E−15	2.64E−12	33.85	− 3.76	1.10E−16	2.47E−14	16.68	− 3.39	2.11E−10	2.75E−08
ASV214	*Alloprevotella tannerae*	13.26	− 3.89	4.01E−18	1.08E−15	4.83	− 2.65	1.50E−10	2.19E−08	7.97	− 3.31	1.85E−14	3.11E−12	9.76	− 1.43	0.0070	0.0370
ASV147	*Prevotella_7 melaninogenica*	13.55	− 3.96	7.15E−18	1.44E−15	5.64	− 2.89	2.13E−11	3.83E−09	10.59	− 3.76	1.47E−16	3.05E−14	6.86	− 3.78	2.74E−14	8.05E−12
ASV461	*Capnocytophaga granulosa*	3.96	− 2.12	3.76E−09	1.73E−07	2.39	− 1.52	5.91E−06	0.0002	3.81	− 2.20	2.70E−09	2.50E−07	2.92	− 2.37	1.86E−09	1.68E−07
ASV490	*Solobacterium moorei*	5.41	− 2.10	5.24E−09	2.17E−07	4.95	− 2.16	1.15E−08	1.12E−06	4.12	− 1.81	2.20E−07	1.09E−05	2.55	− 1.50	4.98E−05	0.0009
ASV426	*Abiotrophia*	5.97	− 1.34	3.92E−05	0.0004	5.04	− 1.21	0.0005	0.0063	5.91	− 1.43	2.50E−05	0.0005	4.33	− 1.35	0.0004	0.0049
ASV127	*Granulicatella*	38.59	− 1.71	4.24E−06	6.87E−05	33.46	− 1.66	3.14E−05	0.0007	31.56	− 1.51	7.21E−05	0.0012	22.75	− 1.43	0.0019	0.0140
ASV291	*Streptococcus*	4.41	− 1.94	1.97E−07	4.95E−06	3.45	− 1.77	2.36E−06	8.60E−05	2.62	− 1.25	0.0003	0.0038	2.36	− 1.54	8.04E−05	0.0013
ASV463	*Streptococcus*	7.72	− 2.00	1.52E−07	4.09E−06	5.66	− 1.72	7.36E−06	0.0002	7.51	− 2.09	1.89E−07	9.79E−06	7.61	− 1.32	0.0072	0.0380
ASV434	*Gemella*	5.93	− 1.78	1.99E−07	4.95E−06	5.33	− 1.79	6.24E−07	3.09E−05	5.39	− 1.76	5.05E−07	2.23E−05	4.06	− 1.82	1.69E−05	0.0004
ASV290	*Selenomonas sputige*	11.13	− 1.27	0.0009	0.0057	11.79	− 1.52	0.0002	0.0036	6.82	− 1.03	0.0066	0.0464	10.22	− 1.74	0.0006	0.0065
ASV421	*Veillonella*	4.09	− 1.96	1.32E−07	3.68E−06	4.13	− 2.16	3.13E−08	2.51E−06	2.25	− 1.14	0.0007	0.0082	1.91	− 1.21	0.0012	0.0100
ASV129	*Veillonella*	10.82	− 2.17	4.08E−07	9.27E−06	7.00	− 1.68	9.08E−05	0.0016	9.73	− 2.15	1.16E−06	4.23E−05	1.89	1.40	0.0005	0.0050
ASV506	*Veillonella dispar*	4.31	− 2.28	4.65E−09	1.97E−07	2.15	− 1.37	6.95E−05	0.0013	2.57	− 1.62	7.34E−06	0.0002	1.44	− 0.96	0.0055	0.0301
ASV186	*Veillonella atypica*	8.62	− 2.45	4.22E−09	1.89E−07	6.59	− 2.24	1.21E−07	7.60E−06	3.46	− 1.16	0.0015	0.0162	3.22	− 1.50	0.0004	0.0046
ASV180	*Veillonella parvula*	14.80	− 2.82	1.21E−10	7.22E−09	9.53	− 2.37	1.15E−07	7.42E−06	8.72	− 2.17	3.47E−07	1.66E−05	6.89	− 2.40	1.91E−06	5.46E−05
ASV694	*Leptotrichia*	2.08	− 1.14	0.0004	0.0026	1.69	− 0.93	0.0031	0.0278	1.62	− 0.83	0.0058	0.0428	1.57	− 1.17	0.0012	0.0100
ASV1007	*Fusobacterium*	2.47	− 1.28	5.46E−05	0.0006	2.09	− 1.15	0.0003	0.0036	1.77	− 0.84	0.0042	0.0344	1.64	− 1.08	0.0014	0.0115
ASV289	*Leptotrichia*	5.09	− 2.49	1.85E−10	1.06E−08	5.38	− 2.81	1.15E−11	2.24E−09	3.72	− 2.19	9.41E−09	7.80E−07	3.31	− 2.59	1.77E−09	1.68E−07
ASV179	*Leptotrichia*	12.94	− 3.86	6.10E−18	1.41E−15	6.07	− 2.97	2.00E−12	5.19E−10	11.16	− 3.81	3.33E−17	8.94E−15	5.95	− 3.56	2.19E−14	8.05E−12
ASV868	*Saccharimonadales*	2.55	− 1.45	1.69E−05	0.0002	2.18	− 1.36	5.54E−05	0.0011	1.67	− 0.88	0.0039	0.0331	2.47	− 2.07	4.13E−07	1.47E−05
ASV423	*Candidatus Saccharimonas*	5.79	− 2.01	2.78E−08	8.62E−07	3.79	− 1.52	1.88E−05	0.0005	5.35	− 2.02	3.70E−08	2.43E−06	4.60	− 2.37	2.46E−08	1.60E−06
ASV336	*Saccharimonadales*	5.95	− 2.75	1.09E−11	8.03E−10	5.22	− 2.77	2.80E−11	4.36E−09	4.18	− 2.37	1.57E−09	1.56E−07	2.67	− 2.21	1.19E−07	5.59E−06
ASV596	*Neisseria elongata*	2.67	− 1.52	5.26E−06	8.01E−05	2.35	− 1.49	9.29E−06	0.0003	2.02	− 1.20	0.0002	0.0032	1.52	− 1.09	0.0016	0.0124
ASV66	*Neisseria*	26.06	− 3.07	4.90E−11	3.44E−09	26.15	− 3.31	2.71E−11	4.36E−09	11.05	− 1.93	1.36E−05	0.0003	12.32	− 2.72	3.26E−07	1.20E−05
ASV582	*Haemophilus haemolyticus*	2.68	− 1.53	6.88E−06	0.0001	2.33	− 1.47	2.00E−05	0.0005	2.23	− 1.35	6.22E−05	0.0011	1.61	− 1.22	0.0004	0.0049
ASV360	*Haemophilus haemolyticus*	5.75	− 1.57	1.06E−05	0.0001	4.17	− 1.22	0.0009	0.0100	5.08	− 1.50	4.11E−05	0.0008	5.41	− 1.49	0.0008	0.0079
ASV37	*Enterobacter kobei*	3.75	− 2.03	9.11E−08	2.63E−06	1.93	− 1.15	0.0005	0.0059	1.71	− 0.92	0.0037	0.0320	2.21	− 1.86	4.02E−06	0.0001
ASV154	*Klebsiella michiganensis*	3.79	− 2.05	7.28E−08	2.18E−06	2.89	− 1.82	1.52E−06	6.46E−05	2.74	− 1.68	3.56E−06	0.0001	1.77	− 1.42	0.0002	0.0026
ASV44	*Serratia marcescens*	8.80	− 3.12	2.74E−12	2.11E−10	7.56	− 3.11	9.30E−12	1.97E−09	9.37	− 3.37	3.77E−13	5.62E−11	1.79	− 1.19	0.0012	0.0100
ASV184	*Haemophilus pittmaniae*	9.66	− 3.43	1.73E−15	2.54E−13	1.68	− 0.96	0.0018	0.0185	11.13	− 3.83	1.20E−17	4.02E−15	3.38	− 2.63	7.38E−10	7.87E−08
ASV58	*Klebsiella michiganensis*	9.85	− 3.46	1.46E−14	1.57E−12	3.68	− 2.20	3.68E−08	2.68E−06	16.05	− 4.34	1.11E−18	4.48E−16	6.59	− 3.72	2.31E−14	8.05E−12

DESeq2 statistics for the 75 ASVs that were significantly differentially abundant across all four models described: SI (N = 47) versus No SI (N = 325, with any PHQ-9 total score), Mild depression (N = 128, with PHQ-9 total scores [5–9]), No depression (N = 157, with PHQ-9 total scores [0–4]), or Depressed (N = 40, with PHQ-9 total scores ≥ 10). Normalized base mean counts (baseMean) of the ASV and log2 fold change (log2FC) are provided for each model, and ASVs with |log2FC| ≥ 2 are highlighted in italics (elevated in individuals with SI) or underline (elevated in individuals without SI). *p* values are provided, both with and without adjustment (padj) for false discovery rates (FDR). Full sequences for all ASVs are provided in Supplementary Table 10.

Among the taxa observed at higher abundances in individuals with SI were members of *Actinobacteria, Bacteroidia (Bacteroides dorei, Prevotella melaninogenica), Bacilli (Streptococcus gordonii), Negativicutes (Veillonella atypica, Veillonella dispar, Dialister, and Veillonella), Fusobacteriia (Leptotrichia and Fusobacterium nucleatum), Patescibacteria (Saccharimonadaceae TM7x), Burkholderiales (Neisseria mucosa), Enterobacterales (Klebsiella oxytoca)*. Among those observed more abundantly in individuals without SI (irrespective of no, mild, or moderately severe depression) were members of *Actinomycetales, Bacteroidales (Alloprevotella rava, Tannerella, Porphyromonas, Alloprevotella tannarae), Capnocytophaga granulosa, Solobacterium moorei, Bacilli (Abiotrophia, Granulicatella, Streptococcus, and Gemella), Negativicutes (Selenomonas sputigena, Veillonella dispar, Veillonella atypica, and Veillonella parvula), Fusobacteriia (Leptotrichia, Fosbacterium), Neisseria, and Gammaproteobacteria (Haemophilus haemolyticus, Enterobacter kobei, Klebsiella michiganensis, Serratia marcescens, Haemophilus pittmaniae, and Klebsiella michiganensis)*.

Of the more dominant ASVs (those with a normalized base mean count > 40; Supplementary Fig. 3g) seen in any of the four models, ASVs belonging of *Lautropia, Veillonella*, and *Megasphaera micronuciformis* were among those more abundant in individuals with SI, while ASVs belonging to *Alloprovetella, Porphyromonas, R. mucilaginosa, N. perflava, P. nanceiensis, S. oralis,* and were more abundant in any of the comparative groups without SI. The largest increase in relative abundance in individuals with SI compared to NOSI was observed in ASV509 (*Bacteroides dorei*, 4.015 log_2_ fold change), and ASVs that were the most elevated in NOSI included ASV214 and ASV250 (*Alloprevotella tannerae*), ASV115 (*Alloprevotella*), and ASV147 (*Prevotella melaninogenica*), all log_2_ fold changes > 3.1.

In the NOSI model comparison, *Veillonella* ASV121 and ASV40 were both elevated in individuals with SI. According to NCBI BLAST, these ASVs have 99.8% and 95.0% homology with *Veillonella nakazawae T1-7*. ASV40 was 3.14× higher in individuals with SI versus those without (NOSI), with a normalized base mean of 125. Individuals with SI were 2× more likely to have a non-zero count of at least one of the ASVs, χ^2^ (1, N = 372) = 5.1, *p* = 0.024. While 51.1% of SI had at least one of these ASVs, they were found at 34.2% prevalence in NOSI.

### Differentially abundant closest species and genera in SI after controlling for sex, diet, and sleep

After propensity score matching on sex and the diet confounders were defined, 17 unique species were discovered to be differentially more abundant in the matched controls (NOSI) and 22 species and 21 genera were more abundant in individuals with SI at *p* < 0.1 (Table 4). Among the NOSI controls,18 genera were more abundant compared to those with SI (Table 4).

**Table 4:** Salivary bacterial genera and closest cultured relative species that are differentially abundant in suicidal ideation (SI) after controlling for sex and diet.

Closest relative cultured species	Higher	1:2 (N = 141)		1:3 (N = 188)		Mean (± SD) relative abundance (%)		
		*H*	*p*	*H*	*p*	SI	No SI (N = 94)	No SI (N = 141)
**Bacteroidetes**								
*Prevotella intermedia*	*No SI*	3.95	0.047	3.37	0.066	0.027 ± 0.117	0.088 ± 0.362	0.077 ± 0.313
**Firmicutes**								
*Granulicatella adiacens*	*No SI*	13.43	< 0.001	12.57	< 0.001	0.457 ± 0.732	0.794 ± 0.827	0.806 ± 0.995
*Streptococcus parasanguinis*	*No SI*	4.02	0.045	5	0.025	0.33 ± 0.457	0.598 ± 0.815	0.655 ± 1.46
**Fusobacteriota**								
*Fusobacterium periodonticum*	*No SI*	5.98	0.014	5.63	0.018	0.868 ± 1.223	1.44 ± 1.508	1.341 ± 1.416
**Proteobacteria**								
*Caulobacter vibrioides*	*No SI*	7.99	0.005	5.66	0.017	0.002 ± 0.016	0.011 ± 0.046	0.008 ± 0.038
*Haemophilus influenzae*	*No SI*	4.28	0.039	4.24	0.04	0.04 ± 0.085	0.11 ± 0.234	0.096 ± 0.205
**Spirochaetota**								
*Treponema amylovorum*	*No SI*	3.49	0.062	4.97	0.026	0.001 ± 0.005	0.009 ± 0.039	0.009 ± 0.036
**Actinobacteriota**								
*Bifidobacterium catenulatum*	*SI*	8.17	0.004	4.06	0.044	0.008 ± 0.034	0 ± 0	0.001 ± 0.008
*Collinsella aerofaciens*	*SI*	6.09	0.014	2.16	0.142	0.006 ± 0.026	0 ± 0	0.001 ± 0.004
**Bacteroidetes**								
*Bacteroides dorei*	*SI*	15.45	< 0.001	17.37	< 0.001	0.089 ± 0.357	0.001 ± 0.007	0.001 ± 0.007
**Firmicutes**								
*Lactococcus lactis*	*SI*	6.43	0.011	11.31	0.001	0.252 ± 1.686	0.002 ± 0.015	0.002 ± 0.012
*Ruminococcus bromii*	*SI*	6.09	0.014	5.45	0.02	0.001 ± 0.003	0 ± 0	0 ± 0.001
*Oribacterium sinus*	*SI*	3.91	0.048	3.94	0.047	0.023 ± 0.113	0.008 ± 0.033	0.006 ± 0.028
*Veillonella rogosae*	*SI*	4.79	0.029	3.7	0.054	0.218 ± 1.148	0.104 ± 0.76	0.097 ± 0.643
*Megasphaera micronuciformis*	*SI*	3.19	0.074	4.11	0.043	4.05 ± 3.929	2.617 ± 2.573	2.647 ± 2.846
*Veillonella atypica*	*SI*	5.22	0.022	3.67	0.055	11.17 ± 10.764	7.138 ± 9.555	8.196 ± 10.511
*Selenomonas artemidis*	*SI*	3.95	0.047	2.08	0.149	0.03 ± 0.104	0.007 ± 0.023	0.011 ± 0.038
**Fusobacteriota**								
*Fusobacterium naviforme*	*SI*	4.25	0.039	2.8	0.094	0.01 ± 0.027	0.004 ± 0.02	0.004 ± 0.018
**Proteobacteria**								
*Methylobacterium-Methylorubrum organophilum*	*SI*	3.17	0.075	5.39	0.02	0.001 ± 0.008	0 ± 0.002	0 ± 0.002

Genus	Higher	Matching 1:2 (N = 141)		Matching 1:3 (N = 188)		Mean ± SD relative abundance (%)		
		*H*	*p*	*H*	*p*	SI	no SI (N = 94)	no SI (N = 141)
**Actinobacteriota**								
*Collinsella*	*SI*	6.09	0.014	1.34	0.248	0.006 ± 0.027	0 ± 0	0.001 ± 0.005
*Senegalimassilia*	*SI*	4.03	0.045	2.86	0.091	0.001 ± 0.007	0 ± 0	0 ± 0.002
**Bacteroidete**								
*Bacteroides*	*SI*	12.86	< 0.001	8.63	0.003	0.093 ± 0.36	0.001 ± 0.008	0.003 ± 0.013
**Firmicutes**								
*Megasphaera*	*SI*	3.39	0.066	4.36	0.037	4.133 ± 3.993	2.653 ± 2.599	2.681 ± 2.872
*Lactococcus*	*SI*	6.43	0.011	11.31	0.001	0.254 ± 1.697	0.002 ± 0.015	0.002 ± 0.013
*Clostridium *sensu stricto* 1*	*SI*	4.87	0.027	0.72	0.397	0.008 ± 0.033	0.001 ± 0.004	0.001 ± 0.006
*Agathobacter*	*SI*	7.16	0.007	2.69	0.101	0.007 ± 0.029	0 ± 0.002	0.002 ± 0.01
*Intestinibacter*	*SI*	4.03	0.045	2.86	0.091	0.002 ± 0.008	0 ± 0	0 ± 0.002
*Ruminococcus*	*SI*	4.92	0.027	5.61	0.018	0.001 ± 0.003	0 ± 0.002	0 ± 0.002
**Proteobacteria**								
*Lautropia*	*SI*	4.65	0.031	7.29	0.007	0.436 ± 1.167	0.265 ± 0.798	0.219 ± 0.671
*Methylobacterium-Methylorubrum*	*SI*	3.17	0.075	5.39	0.02	0.001 ± 0.008	0 ± 0.002	0 ± 0.002
*Pseudomonas*	*SI*	6.09	0.014	3.22	0.073	0.001 ± 0.004	0 ± 0	0.001 ± 0.009
**Bacteroidetes**								
*Porphyromonas*	*No SI*	7.31	0.007	9.6	0.002	0.987 ± 1.232	1.742 ± 1.783	1.933 ± 2.092
*Alloprevotella*	*No SI*	5.93	0.015	6.29	0.012	1.831 ± 2.493	2.679 ± 2.517	2.668 ± 2.727
**Firmicutes**								
*Johnsonella*	*No SI*	4.37	0.037	4.43	0.035	0.051 ± 0.114	0.103 ± 0.162	0.098 ± 0.158
*Gemella*	*No SI*	9.43	0.002	8.07	0.005	0.195 ± 0.205	0.402 ± 0.437	0.394 ± 0.479
*Granulicatella*	*No SI*	13.03	< 0.001	12.15	< 0.001	0.528 ± 0.784	1.003 ± 1.048	0.997 ± 1.161
**Fusobacteriota**								
*Fusobacterium*	*No SI*	3.87	0.049	3.51	0.061	1.328 ± 1.567	1.939 ± 1.835	1.811 ± 1.715
**Proteobacteria**								
*Caulobacter*	*No SI*	7.99	0.005	5.66	0.017	0.002 ± 0.017	0.011 ± 0.047	0.008 ± 0.039
*Massilia*	*No SI*	10.83	0.001	8.22	0.004	0 ± 0	0.027 ± 0.148	0.018 ± 0.121

Relative abundances of significant differentially abundant closest-species-relatives in individuals without SI (NOSI) and individuals with SI (SI) after propensity score matching on sex and diet factors. Two models, with 1:2 and 1:3 matching of SI to NOSI, were conducted and the Kruskal Wallis test results on the relative percent abundances presented. All species and genera presented here have a *p* value of < 0.05 in at least one of the two models.

After additionally matching on sleep issues, 26 genera were discovered to be more abundant in controls and 8 were more abundant in individuals with SI (Table 5). Twenty species were more abundant in controls and 14 were more abundant in individuals with SI (Table 5). Several species and genera were consistent across the two propensity score matching paradigms, demonstrating the bacterial association is present with no regard to sleep as a potential confounder. In individuals with no SI, *Granulicatella adiacens, Streptococcus parasanguinis, Fusobacterium periodonticum, Haemophilus influenzae, Caulobacter vibrioides*, and *Treponema amylovorum* were elevated in both models, as were *Alloprevotella, Porphyromonas, Gemella, Fusobacterium, Massilia*, and *Caulobacter*. In individuals with SI, *Megasphaera micronuciformis, Veillonella rogosae, Lactoboccous lactis*, and *Fusobacterium naviforme*, as well as *Megaphaera, Lactococcus*, and Lautropia were elevated in both models.

**Table 5:** Salivary bacterial genera and closest cultured relative species that are differentially abundant in suicidal ideation (SI) after controlling for sex, diet, and the presence of sleep issues.

Closest relative cultured species	Higher	1:2 (N = 141)		1:3 (N = 188)		Mean (+ SD) Relative abundance (%)		
		*H*	*p*	*H*	*p*	SI (N = 47)	No SI (N = 94)	No SI (N = 141)
**Bacteroidetes**								
*Prevotella melaninogenica*	*No SI*	2.66	0.103	3.91	0.048	3.094 ± 3.924	4.084 ± 4.555	4.24 ± 4.55
*Prevotella nanceiensis*	*No SI*	7.45	0.006	7.17	0.007	0.229 ± 0.326	0.362 ± 0.581	0.368 ± 0.568
**Firmicutes**								
*Streptococcus oralis*	*No SI*	3.94	0.047	3.4	0.065	4.356 ± 4.88	6.948 ± 6.834	6.463 ± 6.284
*Granulicatella adiacens*	*No SI*	14.31	< 0.001	11.02	0.001	0.457 ± 0.732	0.924 ± 1.154	0.837 ± 1.058
*Streptococcus parasanguinis*	*No SI*	8.68	0.003	6.44	0.011	0.33 ± 0.457	0.795 ± 1.759	0.688 ± 1.492
*Solobacterium moorei*	*No SI*	7.16	0.007	8.15	0.004	0.043 ± 0.093	0.077 ± 0.109	0.077 ± 0.111
*Dialister pneumosintes*	*No SI*	4.02	0.045	3.4	0.065	0.003 ± 0.006	0.019 ± 0.066	0.016 ± 0.056
*Peptoanaerobacter stomatis*	*No SI*	4.52	0.033	4.07	0.044	0.001 ± 0.004	0.004 ± 0.012	0.004 ± 0.012
*Mycoplasma orale*	*No SI*	5.91	0.015	4.93	0.026	0.001 ± 0.004	0.007 ± 0.025	0.006 ± 0.022
**Fusobacteriota**								
*Fusobacterium periodonticum*	*No SI*	5.6	0.018	7.91	0.005	0.868 ± 1.223	1.39 ± 1.464	1.413 ± 1.485
**Proteobacteria**								
*Enterobacter kobei*	*No SI*	4.66	0.031	2.83	0.092	0.032 ± 0.219	0.243 ± 1.592	0.162 ± 1.302
*Haemophilus influenzae*	*No SI*	5.24	0.022	4.7	0.03	0.04 ± 0.085	0.119 ± 0.239	0.1 ± 0.209
*Caulobacter vibrioides*	*No SI*	6.2	0.013	5.28	0.022	0.002 ± 0.016	0.006 ± 0.02	0.007 ± 0.028
**Spirochaetota**								
*Treponema amylovorum*	*No SI*	4.02	0.045	3.26	0.071	0.001 ± 0.005	0.01 ± 0.04	0.008 ± 0.033
**Bacteroidetes**								
*Bacteroides dorei*	*SI*	12.84	< 0.001	10.91	0.001	0.089 ± 0.357	0.001 ± 0.007	0.006 ± 0.053
**Firmicutes**								
*Megasphaera micronuciformis*	*SI*	4.07	0.044	3.38	0.066	4.05 ± 3.929	2.536 ± 2.815	2.785 ± 3.13
*Veillonella rogosae*	*SI*	4.11	0.043	1.3	0.254	0.218 ± 1.148	0.138 ± 1.033	0.195 ± 1.176
*Lactococcus lactis*	*SI*	6.43	0.011	9.2	0.002	0.252 ± 1.686	0.002 ± 0.015	0.002 ± 0.013
*Lachnoanaerobaculum umeaense*	*SI*	4.62	0.032	0.84	0.36	0.024 ± 0.146	0.004 ± 0.03	0.01 ± 0.052
**Fusobacteriota**								
*Fusobacterium naviforme*	*SI*	3.91	0.048	1.22	0.269	0.01 ± 0.027	0.005 ± 0.021	0.006 ± 0.02
**Proteobacteria**								
*Neisseria flavescens*	*SI*	4.03	0.045	1.38	0.239	0.006 ± 0.028	0 ± 0	0.001 ± 0.01
*Conservatibacter flavescens*	*SI*	4.81	0.028	2.09	0.148	0.003 ± 0.01	0 ± 0.003	0.001 ± 0.004

Genus	Higher	Matching 1:2 (N = 141)		Matching 1:3 (N = 188)		Mean ± SD relative abundance (%)		
		*H*	*p*	*H*	*p*	SI	NOSI (N = 94)	NOSI (N = 141)
**Actinobacteriota**								
*Actinomyces*	No SI	3.99	0.046	3.17	0.075	0.467 ± 0.658	0.686 ± 0.939	0.637 ± 0.852
**Bacteroidetes**								
*Alloprevotella*	No SI	6.59	0.01	7.93	0.005	1.831 ± 2.493	2.673 ± 2.617	2.61 ± 2.557
*Porphyromonas*	No SI	8.55	0.003	9.68	0.002	0.987 ± 1.232	1.914 ± 2.013	1.907 ± 2.044
*Tannerella*	No SI	2.56	0.11	4.1	0.043	0.025 ± 0.06	0.058 ± 0.179	0.053 ± 0.15
**Firmicutes**								
*Granulicatella*	No SI	13.64	< 0.001	10.38	0.001	0.528 ± 0.784	1.149 ± 1.329	1.023 ± 1.211
*Peptostreptococcus*	No SI	3.35	0.067	4.66	0.031	0.198 ± 0.403	0.26 ± 0.423	0.273 ± 0.411
*Parvimonas*	No SI	1.41	0.236	4.01	0.045	0.134 ± 0.277	0.192 ± 0.297	0.212 ± 0.299
*Stomatobaculum*	No SI	4.37	0.037	4.93	0.026	0.138 ± 0.162	0.191 ± 0.196	0.197 ± 0.226
*Gemella*	No SI	7.51	0.006	6.96	0.008	0.195 ± 0.205	0.437 ± 0.545	0.4 ± 0.494
*Solobacterium*	No SI	7.26	0.007	8.27	0.004	0.044 ± 0.094	0.079 ± 0.11	0.078 ± 0.112
*Defluviitaleaceae UCG-011*	No SI	3.85	0.05	2.08	0.149	0.003 ± 0.007	0.003 ± 0.013	0.003 ± 0.012
*Peptoanaerobacter*	No SI	4.52	0.033	4.07	0.044	0.001 ± 0.004	0.004 ± 0.012	0.004 ± 0.012
*Mycoplasma*	No SI	5.66	0.017	4.68	0.031	0.001 ± 0.004	0.008 ± 0.026	0.007 ± 0.022
**Fusobacteriota**								
*Fusobacterium*	No SI	3.96	0.047	5.17	0.023	1.328 ± 1.567	1.948 ± 1.82	1.906 ± 1.806
**Proteobacteria**								
Enterobacter	No SI	4.77	0.029	3.09	0.079	0.092 ± 0.451	0.887 ± 5.516	0.594 ± 4.515
Massilia	No SI	7.09	0.008	5.78	0.016	0 ± 0	0.01 ± 0.036	0.007 ± 0.03
Caulobacter	No SI	6.2	0.013	5.28	0.022	0.002 ± 0.017	0.006 ± 0.021	0.007 ± 0.028
**Firmicutes**								
*Megasphaera*	SI	4.21	0.04	3.57	0.059	4.133 ± 3.993	2.571 ± 2.844	2.825 ± 3.164
*Lactococcus*	SI	6.43	0.011	9.2	0.002	0.254 ± 1.697	0.002 ± 0.015	0.002 ± 0.013
**Proteobacteria**								
*Lautropia*	SI	6.1	0.013	6.59	0.01	0.436 ± 1.167	0.236 ± 0.79	0.241 ± 0.718
*Conservatibacter*	SI	4.81	0.028	2.09	0.148	0.003 ± 0.01	0 ± 0.003	0.001 ± 0.004

Relative abundances of significant differentially abundant closest species-relatives in individuals without SI (NOSI) and individuals with SI (SI) after propensity score matching on sex, diet factors, as before, and additionally on sleep issues. Two models, with 1:2 and 1:3 matching of SI to NOSI, were conducted and the Kruskal Wallis test results on the relative percent abundances presented. All species and genera presented here have a *p* value of < 0.05 in at least one of the two models. Highlighted are the species and genera that were consistent with the previous propensity score matching paradigm, which did not control for sleep, demonstrating the bacterial association is present with no regard to sleep as a potential confound.

*Granulicatella* was the single genus more abundant (log_2_ Fold Change of 0.903, padj = 0.00913) in NOSI_sleep_ controls than those with SI_sleep_, with a base mean of 241.86. Eight species were more abundant in NOSI_sleep_ controls, including *Capnocytophaga leadbetteri*, *Granulicatella adiacens*, *Prevotella melaninogenica*, and *Prevotella intermedia*, while *Megasphaera micronuciformis* was more abundant in SI_sleep_ (log_2_ Fold Change of 1.203, padj = 0.0264) and with normalized base mean counts of 1800.6 and 1564.6 for *P. melaninogenica* and *M. micronuciformis*, respectively, indicating they are among the more dominant bacteria in the saliva ecosystem. See Supplementary Table 8 for the DESeq2 statistics for the significant genus and species.

### Core microbial differences in individuals with and without SI, irrespective of sleep problems

**Figure 2:**
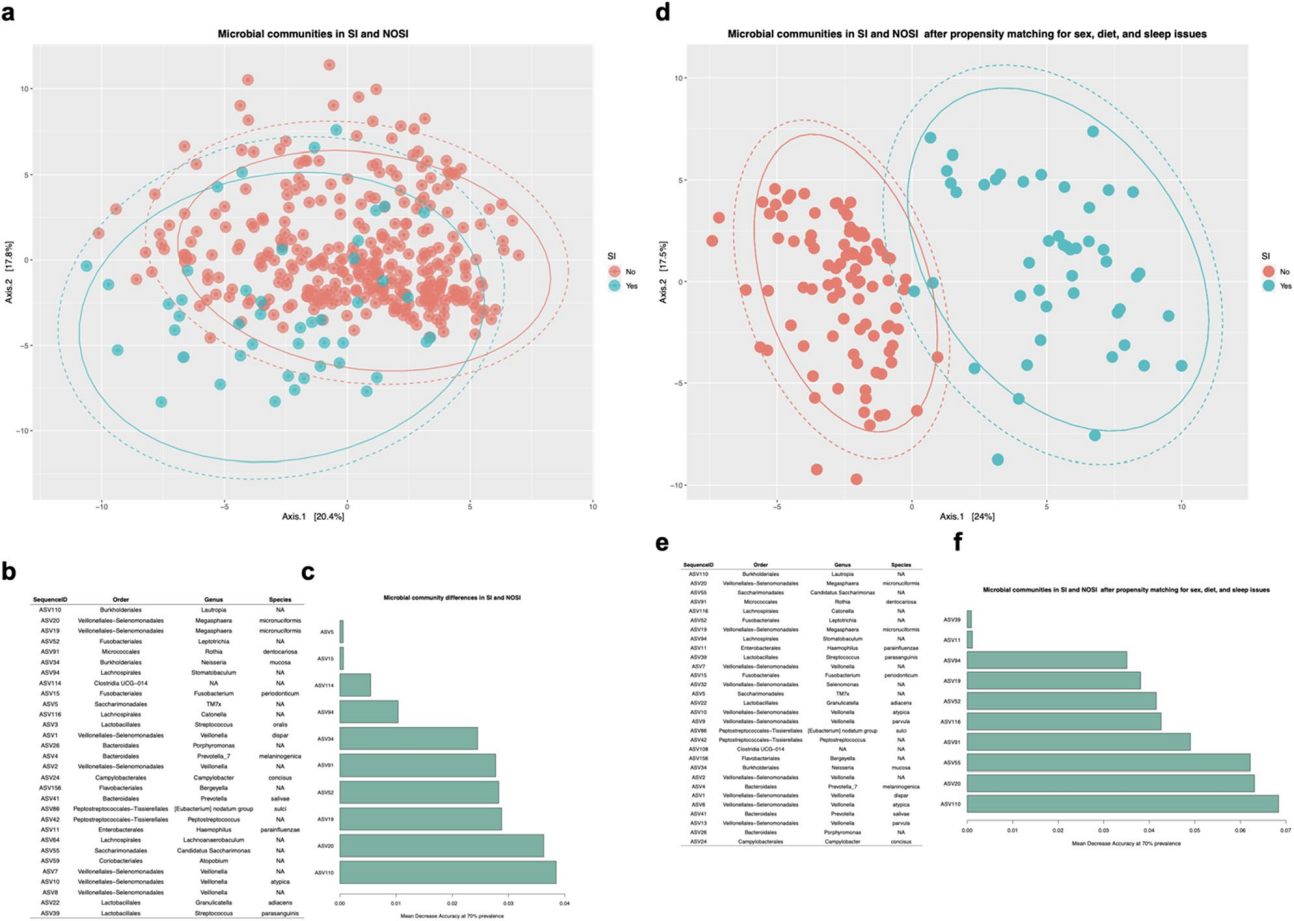
Microbial community prevalence differences in the full cohort (SI vs. NOSI) and in the sleep-restricted cohort (SI_sleep_ vs. NOSI_sleep_). (**a**) Principal coordinates analysis of amplicon sequence variants (ASVs) present at 70% prevalence in either the SI (N = 47) or NOSI (N = 325) in the full dataset. (**b**) Silva taxonomy of the top 30 ASVs contributing to the classification for SI versus NOSI. (**c**) Mean decrease accuracy of the top 10 ASVs for the model. (**d**) Principal coordinates analysis of amplicon sequence variants (ASVs) present at 70% prevalence in either the SI (N = 47) or matched NOSI (N = 94) subjects who were selected by propensity score matching on sex, diet factors, and sleep issues. In this analysis, microbial communities were assessed after removal of the thirteen depressed individuals from the matched NOSI cohort. (**e**) Silva taxonomy of the top 30 ASVs contributing to the classification of SI vs. NOSI after propensity matching. (**f**) Mean decrease accuracy of the top 10 ASVs for the model. Full sequences for the ASVs are provided in Supplementary Table 10.

Within the full cohort, at a threshold of 70% prevalence, representing 39 unique ASVs and 7,570,711 sequences, an out of bag error rate of 0% was observed for SI classification (Fig. 2a). *Lautropia* (ASV110), *Megasphaera micronuciformis* (ASV20, ASV19), *Candidatus Saccharimonas* (ASV55), *Rothia dentocariosa* (ASV91), *Catonella* (ASV116), *Leptotrichia* (ASV52), *Stomatobaculum* (ASV94), *Haemophilus parainfluenzae* (ASV11), and *Streptococcus parasanguinis* (ASV39) were among the top ASVs contributing to the classification at 70% prevalence (Fig. 2b,c).

The same analysis was performed within the cohort matched on sex, diet (consumption of fruit daily, pasta or rice at least five times weekly, nuts at least twice weekly), and whether sleep issues were present, to assess whether similar patterns in the microbiota were observed when holding sleep issues constant. At a threshold of 70% prevalence, 38 ASVs and 2,868,689 resultant sequences were retained with an out-of-bag error rate of 0% for the classification of SI when considering only those individuals with sleep issues (Fig. 2d). The most important ASVs for the classification are listed (Fig. 2e) and include members of *M. micronuciformis, Rothia dentocariosa, F. periodonticum, P. melaninogenica, C. concisus, P. salivae, Eubacterium nodatum group sulci, H. parainfluenzae, V. atypica, G. adiacens, S. parasanguinis* and undetermined species of *Lautropia, Stomatobaculum, Saccharimonadales TM7x, Catonella, Porphyromonas, Bergeyella, Peptostreptococcus, Lachnoanaerobaculum, Candidatus Saccharimonas, Atopobium*, and *Veillonella*, with the mean accuracy for the top 10 most important ASVs provided in Fig. 2f.

Removal of the thirteen individuals from the matched NOSI_sleep_ cohort with the highest PHQ-9 scores (five subjects with moderately severe to severe PHQ-9 total scores and eight subjects with moderate PHQ-9 total scores) did not significantly change the classification error. As with the original model, the top ten ASVs at 70% prevalence included ASV19, 20, 110, 55, 52, 94, 116. However, *Granulicatella adiacens* (ASV22), *Veillonella* (ASV7), and *Streptococcus oralis* (ASV3) comprised the remaining ASVs with the highest contribution to this model in place of *Rothia dentocariosa* (ASV91), *Haemophilus parainfluenzae* (ASV11), and *Streptococcus parasanguinis* (ASV39).

### Salivary microbiota abundances are associated with HLA haplotypes linked to SI

Several notable bacterial species were associated with the HLA haplotypes linked to increased SI incidence in this cohort. Bacterial mean relative abundances are provided in Supplementary Table 9.

In subjects with **DQ2.2 and DQ2.5**, we observed higher relative abundances of *M. micronuciformis* ($\overline{x}$_Not DQ2.2 and DQ2.5_ = 2.85 and $\overline{x}$_DQ2.2 and DQ2.5_ = 4.60, *p* = 0.026) and 9× higher counts of *Veillonella rogosae* ($\overline{x}$_Not DQ2.2 and DQ2.5_ = 0.135 and $\overline{x}$_DQ2.2 and DQ2.5_ = 0.994, *p* = 0.001). *M. micronuciformis* was also elevated in those who were not DR4 and DQ8 (3.1% relative abundance). In these individuals, 2–4× higher relative abundances of *Haemophilus haemolyticus* and *Haemophilus pittmaniae* were also observed.

Those who were **DR4–DQ8** had elevated relative abundances of *Enterobacter kobei* ($\overline{x}$_Not DR4–DQ8_ = 0.029 and $\overline{x}$_DR4–DQ8_ = 1.395, *p* = 0.013) and *Klebsiella aerogenes* ($\overline{x}$_Not DR4–DQ8_ = 0.078 and $\overline{x}$_DR4–DQ8_ = 3.3056, *p* < 0.0001). Moreover, both bacterial species had a mean abundance of 0 in individuals with DQ2.2 and DQ2.5. A 1.5-fold elevation of *Haemophilus pittmaniae* and nearly fourfold elevation of *Veillonella rogosae* were observed in the absence of DR4–DQ8.

In subjects homozygous for DRB1*03, higher abundances of *P. melaninogenica* were observed compared to heterozygotes ($\overline{x}$_DRB1*03 homozygotes_ = 8.731 and $\overline{x}$_DRB1*03 heterozygotes_ = 4.025, *p* = 0.032) as well as in those who did not carry DRB1*03 ($\overline{x}$_Not DRB1*03_ = 3.892, *p* = 0.018). Those who were homozygous for DPA1*01 displayed higher abundances of *Rothia mucilaginosa* than heterozygotes ($\overline{x}$_DPA1*01 homozygotes_ = 1.536 and $\overline{x}$_DPA1*01 heterozygotes_ = 0.9458, *p* = 0.036). *Neisseria mucosa* was elevated in DRB1*15 homozygotes, both when compared to heterozygotes ($\overline{x}$_DRB1*15 homozygotes_ = 1.822 and $\overline{x}$_DRB1*15 heterozygotes_ = 0.454, *p* = 0.003) and when compared to those without the allele ($\overline{x}$_Not DRB1*15_ = 0.619, *p* = 0.007).

The mean relative abundance for *Enterobacter kobei* was observed at 0.0% but 0.35% in those who did not have the haplotype (Supplementary Table 9). Likewise, *Serratia marcescens* was not observed in DRB1*15 homozygotes and was present at 0.90% in those who did not have DRB1*15 and at 0.97% in those with only one allele.

**Figure 3:**
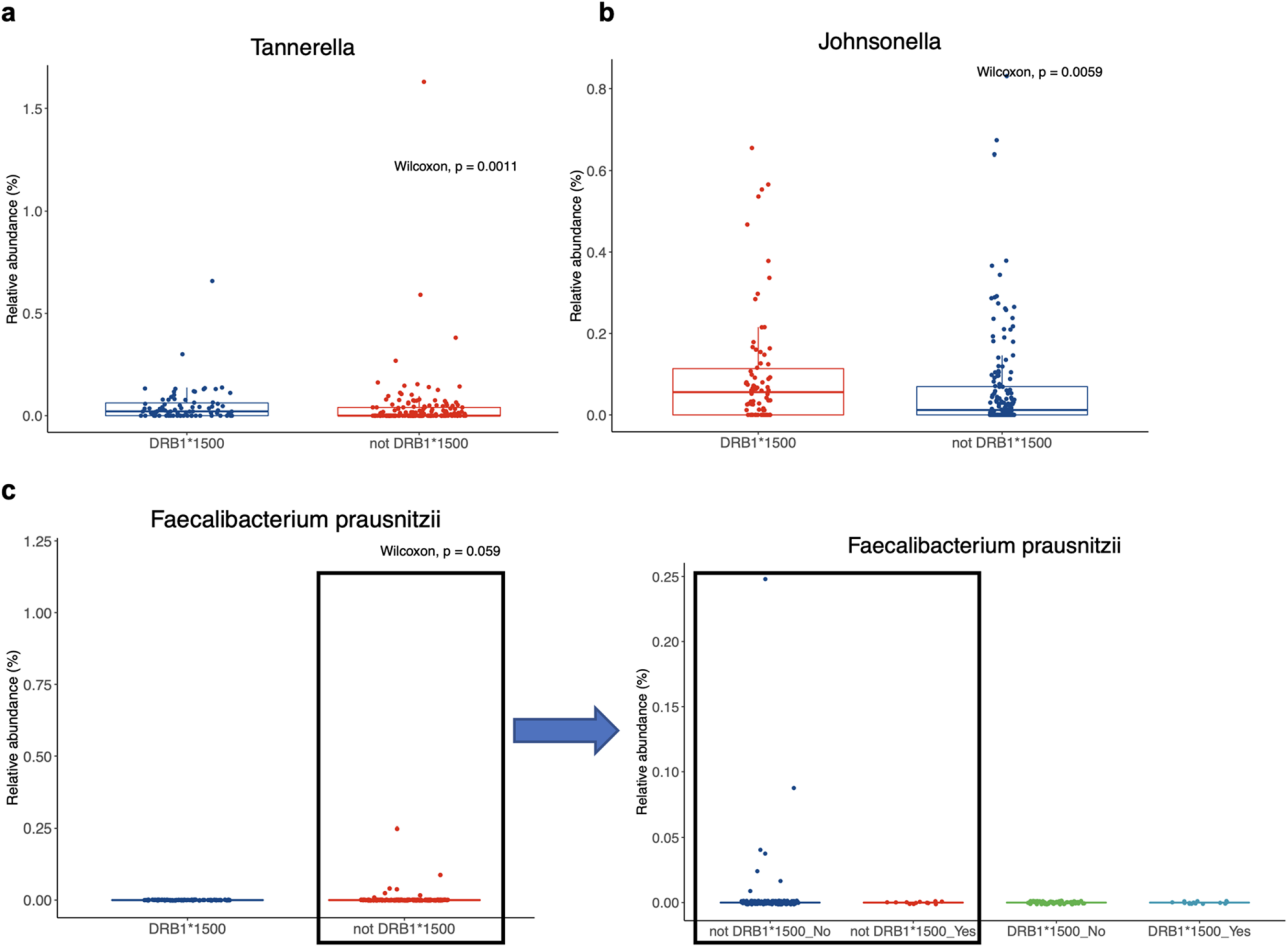
Selected genera and species that are associated with DRB1*1500 genotype and increased incidence of suicidal ideation (SI). Relative abundances of (**a**) *Tannerella* and (**b**) *Johnsonella* were compared across DRB1*1500 groups after controlling for sex, diet factors, and sleep using propensity score matching in a 1:2 ratio of those with or without DRB1*1500 (n = 156). (**c**) Relative abundance of ASVs with a closest cultured relative of *Faecalibacterium prausnitzii*. The two genotype groups are further categorized based on SI endorsement (Yes/No) at right. This species was not observed in any individual endorsing SI and only ever present in those without SI and not harboring the DRB1*15 allele. For all taxa at (**a**–**c**), significance was calculated with a Wilcoxon test statistic between those with or without the DRB1*15 allele.

Although at low abundance, after controlling for sex, dietary habits (e.g., daily consumption of fruit, regular consumption of nuts, dining at a fast-food hamburger restaurant more than once weekly, consumption of pasta or rice every day or at least five times a week), and sleep issues, several genera and species were significantly differentially abundant between those who were DRB1*15 (N = 78) and those who were not (N = 211). Of note, *Tannerella* was observed at significantly higher counts in those with DRB1*15, excepting one outlier who reported to eat sweets frequently (more than three times daily) and pasta or rice more than five times a week (Fig. 3a, *p* = 0.0011). *Johnsonella* was also observed higher frequency in those with DRB1*15 (Fig. 3b, *p* = 0.0059). *Faecalibacterium prausnitzii*, although rarely observed in the saliva, was not present in *any* individual endorsing SI, nor any individual with DRB1*15, both in the full cohort (N = 289) and when controlling for sex, diet, and sleep in a 2:1 propensity score match (N = 234, Fig. 3c).

### Salivary bacteria associated with SI after additionally controlling for HLA genetics

**Figure 4:**
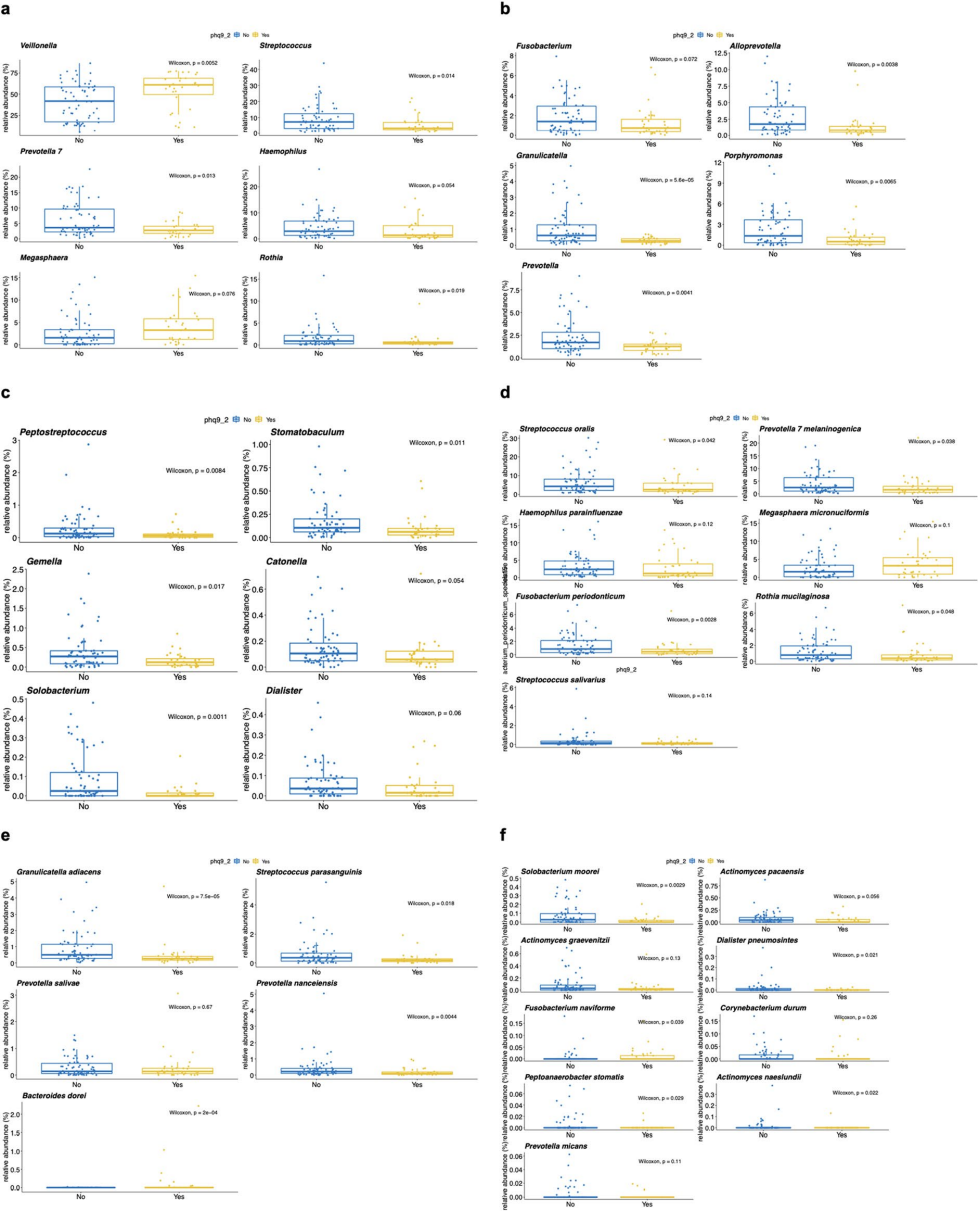
Differentially abundant genera and species in individuals with suicidal ideation (SI) vs individuals without SI (NOSI) after controlling for sex, diet, sleep, and HLA genetics. Wilcoxon statistics (N = 93, SI = 31, NOSI = 62) for relative abundances compared between SI and NOSI according to a 1:2 nearest neighbor selection using propensity score matching paradigm on sex, five dietary habits, presence of sleep issues, and HLA genetics at DRB1*15, DQA1*01, DQB1*03, DPB1*05, and A*30 (haplotypes that were earlier identified to either carry increased risk or appear to be protective for SI in the cohort). Relative abundance (%) is on the y axis. Significant results are presented here at the genus (**a**–**c**) and species (**d**–**f**) taxonomic ranks.

After controlling for sex, dietary habits, presence of sleep issues, and HLA genetics at DRB1*15, DQA1*01, DQB1*03, DPB1*05, and A*30, notable relative abundance differences were discovered in 17 genera and 21 species (Fig. 4). Fifteen genera were significantly more abundant in the NOSI controls, including *Streptococcus, Prevotella, Haemophilus, Rothia, Fusobacterium, Alloprevotella, Granulicatella, Porphyromonas, Peptostreptococcus, Stomatobaculum, Gemella, Catonella, Solobacterium*, and *Dialister* (Fig. 4a–c). *Granulicatella* and *Rothia* were rarely observed at an abundance > 1% in individuals with SI. The saliva microbiome was heavily dominated by *Veillonella* in individuals with SI (median ± SD relative abundance of 60.9% ± 21.1) compared to the NOSI matched controls (41.8% ± 22.2). *Megasphaera* was also significantly higher in individuals with SI.

Excepting *Bacteroides dorei, Megasphaera micronuciformis*, and *Fusobacterium naviforme*, all the identified significant species were higher in abundance in the matched NOSI controls (Fig. 4d–f). *Streptococcus oralis, Prevotella melaninogenica, Haemophilus parainfluenzae, Fusobacterium periodoncitum, Rothia mucilagniosa, Streptoccocus salivarius, Prevotella salivae*, and *Prevotella nanciensis* were all higher in NOSI matched controls, and *Graulicatella adiacens* and *Streptococcus parasanguinis* were rarely observed at an abundance > 1% in individuals with SI. Although generally less abundant in the saliva, *Actinomyces pacaensis* and *Actinomyces graevenitzii* were both observed at higher abundance in NOSI controls. *Solobacterium moorei, Dialister pneumosintes, Corynebacterium durum, Actinomyces naeslundii, Peptoanaerobacter stomatis,* and *Prevotella micans* were rarely observed at all in individuals with SI.

## Discussion

**Figure 5:**
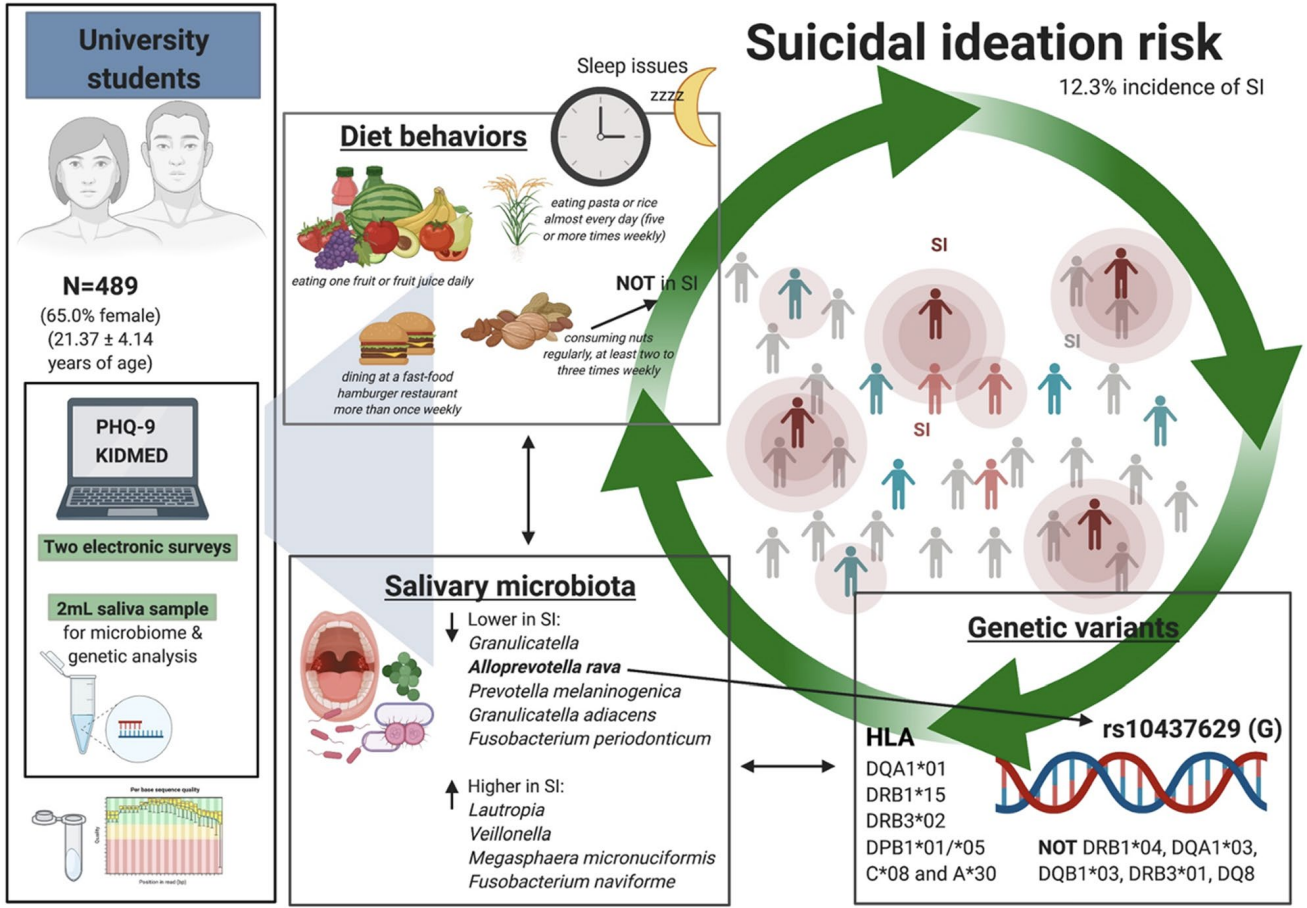
Summary of findings. Here we describe novel associations of diet, genetics, and the saliva microbiome with suicidal ideation (SI) in a cross-sectional study of 489 university students (65.0% female). A 12.3% incidence of SI was observed in this cohort. Students provided a 2-mL saliva sample for microbiome and genetic analysis and completed two brief surveys electronically. Dietary patterns were assessed using the KIDMED survey and SI determined by dichotomized responses to Question 9 of the Patient Health Questionnaire-9 (PHQ-9). Created with Biorender.com.

To our knowledge, this is the first investigation of oral microbiota associations with SI. Suicidal thoughts and behaviors are a strong risk factor for MDD onset^39^, and the converse is also true: MDD increases risk for SI. Discovering links to oral microbiota may help us elucidate novel therapeutic targets for MDD and other neuropsychiatric conditions. Accumulating evidence supports the view that depression is an inflammatory disease, which motivated us to consider HLA genes and salivary microbiota which could contribute to the etiology of SI. Here, we identified novel HLA haplotypes that may be risk indicators or confer protection against SI, as well as salivary microbes that were differentially abundant across these haplotypes (Fig. 5).

Across the analytical approaches applied in this investigation, *Megasphaera micronuciformis* was found to be elevated in individuals with SI. Among the genera that comprise the mixed species signature of periodontitis, confirmed by both culture-dependent and culture-independent methods^31^, is the lactic acid producing genus, *Megasphaera*, which is also elevated in periodontal disease^40^. Although certain *Megasphaera* species may be commensal, such as *Megasphaera* sp. Strain DISK18 which does not possess any virulence determining genes^41^, *M. micronuciformis* has been implicated in various human diseases including bacterial vaginosis^42^ and cancer^43,44^, with evidence to support its role in increasing elevation and cell death^42^. It has been isolated from gut, oral, and female reproductive tract^42,43,45^. *Megasphaera micronuciformis* has been implicated in various cancers, where it is a putative microbial biomarker found commonly on tongue coatings in gastric cancer patients^43^, along with *Selenomonas sputigena ATCC 35185*, and is in elevated abundance in the gut microbiota of locally advanced lung cancer chemotherapy treatment non-responders^44^. *M. micronuciformis* has been shown to elevate inflammatory mediators, induce cytotoxicity, and lead to accumulation of cell membrane lipids in a model of vaginal ecosystems where *Veilloneallaceae* members including *M. micronuciformis* were shown to alter the microenvironment of the cervix^42^. *Selenomonas* was also found at higher abundance in individuals with SI. This is a genus that is elevated in generalized aggressive periodontal disease^46^. Another notable bacterium observed in higher abundances in individuals with SI included *Bacteroides dorei* (log2fold change of 4.0 when compared to all individuals without SI). This bacterium has been implicated as a potential biomarker for future autoimmunity, where it dominates the gut microbiome of infants prior to seroconversion^47^.

Two ASVs that were more likely to be present in individuals with SI and were found at elevated abundance have high shared sequence identity with a newly proposed species of *Veillonella*, *Veillonella nakazawae* T1-7 (AP022321.1 and LC512486.1)^48^. Strain T1–7 produces acetic and propionic acids as fermentation end-products. Its catalase production and partial sequence for *dnaK*, *rpoB*, and citrate synthase (*gltA*) distinguish the species from other members of *Veillonella*, suggesting a novel lineage within the genus. The literature on the oral microbiome and its connection to depression is quite limited, with only one recent publication in this area. The investigation found that *Neisseria* spp. and *Prevotella nigrescens* were more abundant in 40 depressed young adults compared to matched controls^49^. This study did not control for sleep or diet factors, as we have done in the current report, and utilized a six-item instrument adapted from a diagnostic interview which did not capture suicidal behaviors or SI. We controlled for sleep and diet factors, and found decreased abundance in individuals with SI, while the authors found slightly increased abundance in their depression cohort.

Saliva is a major nutrient source for the surface coating of the tongue, contributing glycoproteins, amino acids, and peptides. Some bacteria including *Actinomyces*, *Veillonella*, and *Fusobacterium* are capable of degrading short-chain fatty acids into ammonia, sulfur, and indoles, while the latter two and *Lactobacillus* are also capable of lactate conversion into weaker acids, which can neutralize acid in the mouth^50^. Although *Fusobacterium periodonticum* and *Fusobacterium* as a genus were both overall found in greater abundance in NOSI, one *Fusobacteriaceae* species, *Fusobacterium naviforme*, was found higher in individuals with SI. *Veillonella atypica* was observed in elevated abundances in individuals with SI. In the present report, two *Lactobacilliaceae* species, *G. adiacens* and *S. parasanguinis*, were observed at higher abundance in the absence of SI. All associations occurred after propensity matching on sex and diet, and irrespective of sleep as a potential confounder.

Among the bacteria associated more with the microbiome of the controls rather than those with SI were *Granulicatella*, *R. mucilaginosa*, *Haemophilus parainfluenzae*, *Streptococcus*, and *N. perflava*, which is considered a commensal. These findings are consistent with the current literature investigating the healthy oral cavity. *R. mucilaginosa* is a Gram-positive, facultative anaerobe with weak catalase activity and is a common resident of the natural microbiota of the upper respiratory tract and oropharynx^51,52^. *R. mucilaginosa* was detected at 3.41% of the aerobic microbiota of the human oral cavity and has been isolated from pit, fissure, buccal surface plaque, buccal membrane surface, soft tissues of the oral cavity, and the tongue dorsum, where predominantly resides^52^. In a recent WGS analysis of healthy individuals, *H. parainfluenzae* was found to be the most abundant species in the genus, as were *P. melaninogenica, N. subflava*, and *R. dentocariosa* of their respective genera, while Streptococci were the most abundant genus in the oral cavity^53^. *Haemophilus*, *Streptococcus* and *Pseudomonas* were found to be present on the tongue only in low abundance in patients with liver carcinoma and were more abundant in controls^54^. Likewise, *Granulicella* was significantly elevated in lung cancer chemotherapy responders versus non-responders^33^. Here *Granulicatella*, specifically *Granulicatella adiacens*, was characteristic of individuals without SI all major comparative approaches: (1) SI versus all individuals without SI (NOSI); (2) SI_sleep_ versus NOSI_sleep_ when assessing only those with sleep issues, irrespective of the inclusion or exclusion of NOSI individuals with moderate to moderately severe PHQ-9 scores; and (3) using propensity score matching on sex and diet variables, as well as sleep. Another Lactobacillales bacterium, *Streptococcus parasanguinis*, was discovered to be less abundant in individuals with SI, even after matching on sex, diet, and sleep. *Streptococcus parasanguinis* is a commensal bacterium of the oral cavity and gut^55^ and an early colonizer of the oral cavity^56^ with high prevalence. It possesses the ability to adhere to the oral cavity and bind to other bacteria directly due to its long peritrichous fimbrial surface structures^57^. Importantly, *S. parasanguinis* demonstrates antimicrobial properties in the oral cavity and has been shown to inhibit periodontopathogen growth in vitro through the production of hydrogen peroxide as the primary mechanism^58^, playing a role in the etiology of dysbiotic oral disease.

Increasing evidence of autoimmunity or immune dysfunction being implicated in neurobiological, neurodevelopmental and neuropsychiatric disorders, such as in schizophrenia^14^, motivated us to investigate whether HLA genes encoding proteins that regulate the immune system are associated with increased SI risk or protection and to explore bacterial associations with those haplotypes. Whether HLA genes are involved in psychiatric and neurodevelopmental disorders is not established, with contrasting conclusions. The role of HLA in depression is not clear or may have a moderate effect^20^. HLA genes or C4 complement loci^59,60^ have been implicated in intellectual disability, autism^15–19^, and schizophrenia^61^. Although the haplotype risk is inconclusive^62^, molecular mechanisms involving excessive complement activity may explain in part the synaptic reduction in schizophrenia^61^. Most recently, in a large dataset of over 65,000 individuals participating in the Danish Integrative Psychiatric Research Consortium, Nudel et al. were unable to replicate reported associations but found protective effects of DPB1*1505 on autism and intellectual disability^15^.

Here, those with DQ2.2 and DQ2.5 alleles were more than three times as likely to endorse SI, as were those who did not carry DR4–DQ8. None of the 55 subjects carrying the homozygous DR4–DQ8 allele expressed SI but was found in 51 of the 235 subjects (21.7%) without SI. DR4–DQ8 is associated with multiple autoimmune diseases including celiac disease and type 1 diabetes^63^. We are not aware of any connections between DR4–DQ8 and mental health. There are reports of connections between celiac disease and cognition, but these are controversial. The DR4–DQ8 positive association with no SI and celiac disease might predict that celiac disease would not be associated with mental health challenges. The DRB1*15 allele doubled the chances of SI in this cohort. DRB1*15 increases risk for multiple sclerosis^63–66^. A previous connection with a nervous disorder is consistent with a role for DRB1*15 in SI. DRB1*04 is negatively associated with schizophrenia^15^. DPB1*15 may be protective against intellectual disability and autism^15^. The associations in this cohort provide additional evidence that HLA may contribute to depressive etiologies.

In this work, we also assessed SNPs that have been associated with suicidality in published GWAS studies. Of the fourteen SNPs we tested, rs10437629 was significantly associated with incidence of SI in this cohort. This SNP has been previously identified as associated with attempted suicide^6^. We found that the homozygous GG or heterozygous AG alternative alleles were positively correlated with SI. This SNP is located within an intron of the gene coding for the membrane bound KIAA1549L protein, which suggests that the effect of this mutation may be regulatory. Furthermore, the KIAA1549L protein is enriched in the brain and parathyroid gland^67^. Succinate-producing *Alloprevotella rava* was only found in those without SI and only in those homozygous for rs10437629 AA alleles (Fig. 2b). Succinate has been shown to be produced by gut bacteria and is a known signaling molecule to the brain. Succinate has long been known as a short chain fatty acid that assists in glucose oxidation in the brain and in brain respiration^68,69^. Application of succinate to the brain can improve cerebral metabolism after traumatic brain injury^70,71^. Moreover, succinic acid is among several plasma metabolite neurotransmitters proposed to have diagnostic capability in MDD, found at significantly reduced levels in plasma of individuals with MDD in a metabolomics study^72^. Succinate levels have been reported to be 2.3 times higher in the saliva of males compared to the saliva of females^73^. The mechanism for higher levels of succinate in male versus female saliva is unknown. This is consistent within our cohort, where females were 1.94 times more likely to express SI than males.

Investigating suicidal behaviors at the university affords the opportunity to intervene in at-risk students prior to perceived and anticipated stress events, such as mid-term examinations, in a population where the incidence of suicidal ideation is high. Academic stress has been associated with increased interleukin-1B^74^, plaque^75^, and gingival inflammation^76^ and it is unknown whether the bacteria identified here mediate these associations. The microbiota may provide a potential therapeutic target for brain health and neurological disorders given their role in neuroinflammation^25^. Although we did not assess inflammatory markers, the present study identifies specific bacteria in the mouth that appear to occur more frequently or at higher abundances in individuals with SI and bring to question the mechanism by which such bacteria might confer risk or protection against these phenotypes. Chronic low-grade inflammation may increase risk for MDD. Basal and LPS-stimulated inflammatory markers predicted MDD symptoms over nine years in the Netherlands Study of Depression and Anxiety (NESDA) cohort of MDD patients^77^. Environmental selection of microbiota based on resilience and diversity may be partly responsible for the rise of autoimmune and inflammatory disorders^78^ but could also contribute to the etiology of depression. Understanding how these bacteria promote inflammation may give mechanistic insight into the diatheses of suicide, offering opportunity to characterize an individual’s susceptibility to stressors, including precipitating and perpetuating factors.

This work is the first to report associations of the oral microbiome with recent SI. It is important to note that the PHQ-9, upon which these results are based, only assessed for SI in the past 2 weeks. As such, it is possible that some individuals in the “No SI” control group had a history of SI or self-harm, which could alter the interpretation of results. History of self-harm was not addressed in this investigation, which would help elucidate whether the findings are specific to SI or to suicidal behaviors in general. Metrics such as prior history of SI and suicidal behaviors, antidepressant use, and comorbidities would benefit future extension and confirmation of these findings. Drug by gene interactions may increase risk of SI as a result of antidepressant treatment^10^. Data on antidepressant use must be included in future cohorts. Likewise, although several significant confounds of the microbiome, such as sex, sleep, and diet, were accounted for in this investigation, other data were not obtained, such as geography or ethnicity of the subjects. Because the present analyses are cross-sectional, we cannot speak to the chronology or causality of the factors that we have identified as potential risk indicators or being associated with increased incidence of SI. In this investigation, we assigned species names based on the closest cultured relative and 100% matching of the ASV using the 16S rRNA V3–V4 methodology. To be more confident in the precise taxonomy of the bacteria, metagenomic sequencing is warranted to interrogate bacterial functions and/or other sequencing methods such as the rrn operon to obtain longer reads. Investigating the neurophysiological consequences of these microbes in the pathology of MDD and suicidal behaviors will require longitudinal cohorts and further study on the role in precipitating and perpetuating inflammation.

Here, we identified novel connections between diet, genetics, and saliva microbiome composition in suicidal ideation in young adults, expanding on the existing evidence supporting the diathesis of suicidality. To our knowledge, this is the first study to demonstrate a role for genetic and salivary bacterial interactions in the etiology of suicidal ideations. Our work warrants further investigation into the role that rs10437629 and HLA play in SI, and whether these genotypes influence suicidality risk via the saliva microbiota or independently. Evidence supports the notion that depression is an inflammatory disease^79^, with at least one-quarter of patients with depression demonstrating low-grade inflammation^80^. Elucidating how the oral microbiota mediate or contribute to the inflammatory and metabolic status of the oral milieu may inform novel interventions or preventions for MDD and SI, especially given the emerging and important connection between the mouth and brain.

## Methods

### Study design

Undergraduate and graduate students taking Microbiology and Cell Science courses at the University of Florida voluntarily enrolled in the study between August 2018 and February 2020. The study was approved by and carried out in accordance with the University of Florida Institutional Review Board (IRB), with study approval IRB study #201801744. All methods were carried out in accordance with relevant guidelines and regulations.

Participants were recruited from Microbiology & Cell Science courses as approved by the IRB. The cohort was comprised of 489 total students of whom 318 were female (65.0%) and 171 were male; the average age was 21.37 ± 4.14 years. After providing their informed consent, participants completed the Mediterranean Diet Quality Index (KIDMED^81^) and the Patient Health Questionnaire (PHQ-9^38^) electronically through Research Electronic Data Capture (REDCap)^82^. The KIDMED was initially developed for children and adolescents, but has been validated in college students as a measure of Mediterranean diet adherence, with adequate test–retest reliability^83^. The PHQ-9 is widely used in clinical and research settings, with a sensitivity of 88% (potentially higher^84^) and specificity of 88% for identifying major depression in respondents scoring ≥ 10. The 1-factor structure of the PHQ-9 has been validated in a sample of 857 U.S. college students, suggesting equivalent assessment across sex and racial/ethnic groups. Subjects provided a 2 mL saliva sample concurrently with the survey using the IsoHelix GeneFix DNA/RNA Collection kit (IsohelixTM DNA/RNA GFX-02 2 mL; Cell Projects Ltd., Harrietsham, UK), which stabilizes DNA and RNA long term at ambient temperature. Students who screened positive for suicidal ideation (SI) based on a non-zero response on question 9 of the PHQ-9 as well as those who scored > 19 on the PHQ-9 were immediately referred to the Counseling and Wellness Center at UF and those students who scored in the moderate to moderately severe range (10–19) were informed of mental health resources.

Depression levels were calculated from the PHQ-9 total score as previously documented by Kroenke et al.^38^ Binary (zero or non-zero) responses were considered in this manuscript for questions 3 and 9 of the PHQ-9, pertaining to sleep issues and SI, respectively. A non-zero response on PHQ-9 question 9 reflects that the participant denied having had “thoughts that [they] would be better off dead, or of hurting [themselves] in some way” for several days or more over the past 2 weeks^38^. A non-zero response on PHQ-9 question 3 reflects that the participant denied having “trouble falling or staying asleep, or sleeping too much” several days or more over the past 2 weeks.

### Processing of saliva samples

Saliva samples were stored at ambient temperature per the manufacturer’s instructions. These kits are designed to collect a 2 mL saliva sample into a 2 mL mix of proprietary stabilization and lysis buffer pre-filled into the collection kit. The optimized stabilization buffer allows for long-term storage of DNA and RNA at ambient temperature. DNA was extracted and stored at 4C. Extraction was carried out in accordance with the Isolation Protocol for GFX-02 GeneFix saliva samples, with the following revisions: incubation at 37 °C and an increased TE/DNA rehydration step to a duration of 95 min, and omission of purification steps 9–11 since purification was subsequently carried out with Qiagen or the Zymo Genomic DNA Clean & Concentrator kit. DNA was quantified, and 260/280 and 260/230 values were obtained with NanoDrop spectrophotometer (Thermo Scientific, Wilmington, DE). The DNA was then fractionated for 16S rRNA and human genetic analysis.

### 16S rRNA gene PCR and amplicon sequencing

The V3-V4 variable region of the bacterial 16S rRNA gene was polymerase chain reaction (PCR)-amplified with 341F (NNNNCCTACGGGAGGCAGCAG) and 806R (GGGGACTACVSGGGTATCTAAT) with 1 µM final concentration of Illumina fusion, using single-indexed, barcoded primers. Primers, purified with Qubit dsDNA High Sensitivity (Invitrogen, Life technologies Inc., Carlsbad, CA), and quantified with the 1X dsDNA High Sensitivity kit with a Qubit 2.0 fluorometer (Invitrogen, Life Technologies Inc., Carlsbad, CA as we have previously described^85^. Briefly, 50 ng of template DNA for each sample was taken into the PCR reaction. The templates were barcoded and adapted using custom primers during PCR amplification prior to pooling. Standard Illumina Read 1 sequencing/indexing primers were used to produce the amplicons. Each of the standard Illumina barcodes carries 11 unique nucleotides. PCR amplification was carried out with an initial denaturation of 95 °C for 3 min, 25 cycles of 95 °C for 45 s, 53 °C for 30 s, and 75 °C for 90 s, with a final step of elongation at 75 °C for 10 min. Those samples that did not initially amplify were subjected to barcode shotgun PCR. One sample did not amplify with barcode shotgun PCR and thus was excluded from the analysis. Fragment size was verified with gel electrophoresis, and the purified PCR products were quantified using the 1X dsDNA High Sensitivity kit with a Qubit 2.0 fluorometer (Invitrogen, Life Technologies Inc., Carlsbad, CA) via Qubit fluorometer.

PCR products were spin column purified using either a Qiagen kit or the Zymo Genomic DNA Clean & Concentrator kit, and then their concentration was determined using Qubit dsDNA High Sensitivity (Invitrogen, Life Technologies Inc., Carlsbad, CA). Equal masses of 40 ng DNA for each sample were pooled for Illumina MiSeq processing. The three pools of the barcoded, paired-end amplicons were then sequenced using the Illumina MiSeq with 2x300 cycles at UF’s Interdisciplinary Center for Biotechnology Research (ICBR) NextGen DNA Sequencing facility.

### Amplicon processing

Forward and reverse reads were merged using Qiime1 using the join_paired_ends.py script. Demultiplexing was performed either with Qiime1 (split_libraries_fastq.py and split_sequence_file_on_sample_ids.py) or the BBMap pipeline.

The sample inference program DADA2^86^ was employed in R for filtering, trimming, and taxonomic assignment of the amplicon sequence variants (ASVs) with single-nucleotide resolution. DADA2 has demonstrated fewer false positive sequence variants than OTU-based methods, and its resolution of biological differences allows for exact sequence inference. It has been suggested that use of ASVs with exact matching (or 100% identity) is an appropriate method for assigning bacterial species to amplicons generated by high-throughput 16S rRNA techniques^86^. However, species assignments here are based on the closest cultured relatives in the Silva database. Reads shorter than 420 bp were discarded, as were reads that matched against the phiX genome. Amplicons were truncated to remove low quality tails and the 21 bp forward primer was removed, resulting in reads with 399 nucleotides in length. Reads with at least one ambiguous nucleotide were filtered out. Reads were truncated at the first instance of a quality score ≤ 11. The maximum error allowed in a read was set to 1^87^. Error rates were learned by DADA2’s machine learning algorithm. The resulting reads from the four sequencing pools were merged and chimeras removed. Between 95.47 and 98.43% of sequence variants remained in each pool after chimera removal.

In aggregate, the 372 samples for whom we had microbiome data represented 9352 unique ASVs (6125 without chimeras) with 96.83% of the sequence variants remaining after chimera removal. A total of 13,259,769 reads were present in the dataset, with an average of 35,644.54 (median 33,751) reads per sample. The fewest number of reads in a sample was 6506.

Taxonomy was assigned in DADA2 with the assignTaxonomy and addSpecies functions, using the Silva version 138 database^88^. Species-level assignments were exclusively reserved for *exact* matching between the ASVs and reference strains, which is considered optimal for identity thresholds using 16S rRNA sequencing data^89^. Full sequences corresponding to the 6135 ASVs are provided as a reference in Supplementary Table 10, along with the taxonomic assignment from SILVA.

### Human genotyping and HLA imputations

Extracted DNA was purified using either a Qiagen kit or the Zymo Genomic DNA Clean & Concentrator kit (Genomic DNA Clean & Concentrator-10; Zymo Research, Irvine, CA USA) for high yield ultra-pure and large fragment recovery. DNA concentration was determined using Qubit dsDNA High Sensitivity (Invitrogen, Life technologies Inc., Carlsbad, CA). Purified samples were stored at 4 °C. 100 ng of DNA at a concentration of 5 ng/uL were plated and then sequenced using the Axiom Precision Medicine Research Array (PMRA) (Applied Biosystems, ThermoFisher Scientific) against a standard control for microarray analysis according to the PMRA manufacturer’s instructions. The PMRA has a genome-wide imputation grid that covers over 902,560 single-nucleotide polymorphism (SNP) markers. The sequencing was performed according to the protocol described in the Axiom 2.0 Assay Manual Workflow User Guide (Thermo Fisher Scientific Inc.) with the GeneTitan MC Instrument. We generated call codes from the SNP data of the 310 subjects whose samples were sequenced by the PMRA and met quality control cut-offs, using the Axiom Array Suite Software (Thermo Fisher Scientific Inc.).

HLA haplotypes were imputed from the SNP genotype data with the Axiom HLA Analysis Suite version 1.2 (Thermo Fisher Scientific Inc.). This imputation determines the HLA type of 11 major MHC Class I and Class II loci with 2-digit and 4-digit resolution over an extended major histocompatibility (MHC) region that affords accurate HLA typing from SNPs^90^, using a multi-population reference panel and HLA imputation model to infer HLA from Affymetrix genotyping arrays^91^. The HLA*IMP:02 model is tolerant of missing data and performs at 2-digit resolution with an accuracy of over 90% across most ethnicities (and with 95% accuracy at 4-digit resolution on diverse European panels). HLA markers were evaluated at 2-digit resolution.

### Statistical analyses

Continuous variables (age, KIDMED and PHQ-9 total scores) were compared by sex and across depression severity levels using a Mann–Whitney U or Kruskal Wallis test after testing the dataset for normal distribution. Correlations between the sixteen binary dietary habits and HLA haplotypes with recent SI (non-zero scores on PHQ-9:9) were conducted with chi-square tests. These tests were carried out in SPSS version 27 (IBM). All statistical tests were two-tailed.

### HLA Class II DQA1*01, DQA1*03, DQB1*03, and DRB1*015 associations with increased incidence of SI

HLA Class I and Class II haplotype distributions for A*01, A*02, DPA1*01, DPA1*02, DPA1*03, DPA1*04, DPB1*01, DQA1*01, DQA1*01, DQA1*01, DQA1*05, DQB1*03, DQB1*05, DRB1*03, DRB1*04, DRB1*15, DRB5*01, and DRB5*99 were compared between the NOSI and SI groups using a Pearson chi-square statistic in SPSS. For those haplotypes with lower incidence, subjects with 1–2 alleles of the haplotype were combined. Individuals who had at least one allele each of DQA1*02 and DQB1*02 were designated DQ2.2, and those with at least one allele each of DQA1*05 and DQB1*02 were designated DQ2.5. Those who were DQA1*03 and DQB1*03 were designated DQ8, and those with DRB1*04 and DQ8 designated DR4–DQ8.

### Human SNPs associated with increased incidence of SI and subsequent testing of associations with bacterial abundances

There are sixteen single nucleotide polymorphisms (SNPs) assessed by the PMRA and with an EBI trait inclusive of the key word “suicide” or “suicidal.” These SNPs are rs12373805, rs17387100, rs11143230, rs4918918, rs300774, rs358592, rs2462021, rs10437629, rs2610025, rs10748045, rs10448044, rs3781878, rs10854398, rs17173608, rs4732812, and rs7296262. The MAF of each SNP was calculated within the cohort (Supplementary Table 11). Fourteen of the SNPs were observed at a MAF ≥ 0.010, while rs17387100 and rs17173608 were below. The genotypes at these fourteen loci (excluding rs17387100 and rs17173608) were assessed in the cohort with respect to the presence of SI using 3 × 2 and 2 × 2 chi square tests based on dosage and then presence of the minor allele (homozygotes and heterozygotes combined). Kruskal Wallis statistics were then conducted to identify whether and which genera and species were differentially abundant in individuals carrying the risk allele for those SNPs that we found to be associated with SI.

### Bacterial statistical analyses

#### Confounders of the saliva microbiome

To determine which diet features differed in saliva microbial composition and whether there was microbial divergence among those with or without SI, a PERMANOVA based on Bray–Curtis distance matrix was conducted using the adonis function of the vegan R package on the compositionally transformed counts of the 6,135 ASVs that we identified across the 372 samples. The computation was carried out with 1000 permutations. Reads were compositionally transformed using the microbiome R package.

#### Microbiome diversity

Global ecosystem state measures including richness^92^, evenness , and diversity were calculated using the alpha function of the microbiome R package and compared across the N_sleep_ cohort, comprised of subjects with sleep issues only (N_sleep_ = 229 with SI_sleep_ = 43 and NOSI_sleep_ = 186), using a non-parametric Wilcoxon test in ggpubr.

#### Differentially expressed ASVs in individuals with SI

To assess differential expression of the 6,135 ASVs across SI (n = 47) and various depression categories, we first used an RNA-seq based method for assessing a negative binomial distribution by maximum likelihood estimation, DESeq2^93^ which has been adapted for microbiome data^94^. For this approach, the log geometric means were calculated using the unnormalized counts of the 6,135 ASVs and applied a pseudo-count of 1. Independent filtering was applied in the DESeq2 package to optimize the adjusted *p* value by hypothesis weighting^95^.

Four comparative groups were compared against SI using this approach: (1) all subjects without SI (NOSI, n = 325), (2) subjects without SI and with a PHQ-9 total score of 0–4 (no or minimal depression, NODEP, n = 157), (3) subjects without SI and with PHQ-9 of 5–9 (mild depression, MILD, n = 128), (4) subjects without SI and with a PHQ-9 score ≥ 10 (high likelihood of depression, DEP, n = 40). For each model, outliers were replaced and 2076 (NOSI), 1369 (DEP), 1813 (NODEP), 1746 (MILD) ASVs were refitted for differential abundance analyses. A local smoothed dispersion fit was applied to estimate the dispersions, and the Wald test used to assess statistical significance. Prevalence differences were calculated using a chi-square test in a SI (n = 47) versus NOSI (n = 325) comparison.

#### Network analysis of salivary microbiota

Genus-level networks were assessed using a Spearman correlation in MicrobiomeAnalyst^96^. To control for sleep issues, the correlation was carried out with the N_sleep_ restricted cohort (N_sleep_ = 229).

#### Differentially abundant closest species and genera in individuals with SI after controlling for sex, diet, and sleep

ASVs were combined at higher taxonomic ranks using the tax_glom function of phyloseq. 60.7% of ASVs were identifiable with a closest microbiome relative at the species level in SILVA. Of the 560 species observed in the saliva, 177 were found in at least 2% of the cohort and were retained for differential abundance analysis. Of the 227 genera observed in the saliva, 118 were present in at least 2% of the subjects.

To offset potential over-dispersion that could result from application of RNA-seq paradigms to microbiome datasets^97^, differential abundances were also modeled with nonparametric approaches after matching on potential confounders. Propensity score matching was employed using the matchit function of the MatchIt R package to match SI and NOSI samples on confounders of SI or the microbiome: sex, daily fruit consumption, regular consumption of pasta and rice, dining at a fast-food hamburger restaurant more than once weekly, and regular consumption of nuts, since we found that the former and latter are associated with increased risk of SI and the other variables are possible confounders for the saliva microbiome. Propensity score matching was employed at both a 1:2 and 1:3 ratio of SI to NOSI controls. Given that individuals with SI were significantly more likely to report sleep issues on the PHQ-9 (91.5% SI vs. 57.2% NOSI), propensity score matching was again applied to additionally account for sleep differences using a dichotomous classification of response to Q3 of the PHQ-9. In a third model controlling for HLA genetics, propensity score matching was applied on the previously mentioned variables (sex, five dietary habits, and presence of sleep issues) as well as DRB1*15, DQA1*01, DQB1*03, DPB1*05, and A*30 genotype at a 1:2 ratio of SI to NOSI controls. Only individuals with a genotype at all five haplotypes were included in this model, for an N of 93 (SI n = 31, NOSI n = 62).

For the propensity score matching analyses, Kruskal–Wallis or Mann–Whitney tests were conducted in R across the 177 species’ and 118 genera’s percent relative abundances to identify significant differentially abundant features along each of the matching paradigms.

In a restricted dataset comprised only of those individuals who reported sleep issues (N_sleep_ = 229), DESeq2 was run again at the genus and species levels to identify differentially abundant taxa. Of the 227 and 560 genera and species respectively, 75 genera and 219 species passed the internal filtering of the DESeq2 program. These were considered in the final dispersion estimates and model fitting. Those who reported sleep problems and were included in this restricted N_sleep_ dataset.

#### Core microbial differences in individuals with SI after matching on sleep issues

Microbial communities were defined in the SI_sleep_ and NOSI_sleep_ cohorts selected in the 2:1 propensity score match. In the 47 SI_sleep_ and 94 NOSI_sleep_ individuals, a total of 1960 and 2881 unique ASVs were observed respectively. Prevalence thresholds were determined with out-of-bag error rates for the classification of SI and the ASVs comprising the prevalence threshold resulting in 0% error using the PIME package^98^ in R.

#### Salivary species and genera associated with HLA haplotypes linked to SI

With a two-sided means test in SPSS v27 (IBM), the relative abundances of filtered species and genera were compared across the HLA haplotypes linked to SI risk (DQ2.2 and DQ2.5, DQ8, DR4–DQ8, and DRB1*15).

## Supplementary Information

Supplementary Information 1.

Supplementary Information 2.

## Data Availability

The microbiome data and associated metadata used for this work are available from NCBI BioProject ID PRJNA846199. Human genotyping data that support the findings of this study are available from the corresponding author upon reasonable request.
