# Supplementary material for: Saliva microbiome, dietary, and genetic markers are associated with suicidal ideation in university students
Source: Sci Rep. 2022 Aug 22;12:14306. doi: 10.1038/s41598-022-18020-2 (PMC9395396; doi:10.1038/s41598-022-18020-2)
Supplement: Supplementary file 2 — Supplementary Information 2. [file 41598_2022_18020_MOESM2_ESM.docx]

**Saliva microbiome, dietary, and genetic markers are associated with suicidal ideation in university students**

SUPPLEMENTAL MATERIALS

**Supplementary Table 1. Possible confounders of saliva microbiome composition.** PERMANOVA test results after 1000 permutations on the compositional-transformed counts of the 6,135 ASVs identified in 372 subjects (N=47 with suicidal ideation). KIDMED level was computed by total score as published, with poor (scores of <4), medium (4-7), and high (≥8) compliance scores assessed. PHQ-9:2 is the dichotomized (zero or non-zero) response from question 9 of the Patient Health Questionnaire-9 PHQ-9) assessing thoughts of self-harm over the two weeks prior to the survey.

|  | | *R^2^* | *p* |
| --- | --- | --- | --- |
| Sex | | 0.00552 | 0.01598 |
| KIDMED Features | |  |  |
| *Do you eat a fruit or fruit juice every day?* | | 0.00384 | 0.0989 |
| *Do you eat a second fruit or drink a second fruit juice each day?* | | 0.00161 | 0.93407 |
| *Do you have one serving of fruit or cooked vegetables regularly every day?* | | 0.00281 | 0.31968 |
| *Do you have a serving of fresh or cooked vegetables regularly more than once each day?* | | 0.00209 | 0.71129 |
| *Do you consume fish regularly? (at least 2-3 times per week)* | | 0.00245 | 0.49351 |
| *Do you eat at a fast-food hamburger restaurant more than once per week?* | | 0.00387 | 0.09491 |
| *Do you eat pulses (beans, peas, or lentils) more than once per week?* | | 0.00286 | 0.31269 |
| *Do you consume pasta or rice almost every day (5 or more times per week)?* | | 0.00587 | 0.01499 |
| *Do you have cereals or grains (bread, etc.) for breakfast?* | | 0.00278 | 0.36663 |
| *Do you consume nuts regularly (at least 2-3 times per week)?* | | 0.00214 | 0.69231 |
| *Do you skip breakfast (more than 3 times per week)?* | | 0.0038 | 0.1029 |
| *Do you use olive oil at home?* | | 0.00308 | 0.24875 |
| *Do you consume a dairy product for breakfast (yogurt, milk, etc.)?* | | 0.0022 | 0.61938 |
| *Do you regularly eat commercially baked goods or pastries at breakfast (more than 3 times per week)?* | | 0.00205 | 0.73926 |
| *Do you regularly have 2 servings (40g) of yogurt and/or some cheese daily (at least 3 times per week)?* | | 0.00262 | 0.42557 |
| *Do you typically eat sweets or candy several (3 or more) times a day?* | | 0.00349 | 0.15584 |
| KIDMED level (poor, medium, high compliance) | | 0.0045 | 0.6973 |
| PHQ-9:2 Suicidal thoughts in past 2 weeks | 0.00447 | | 0.04695 |


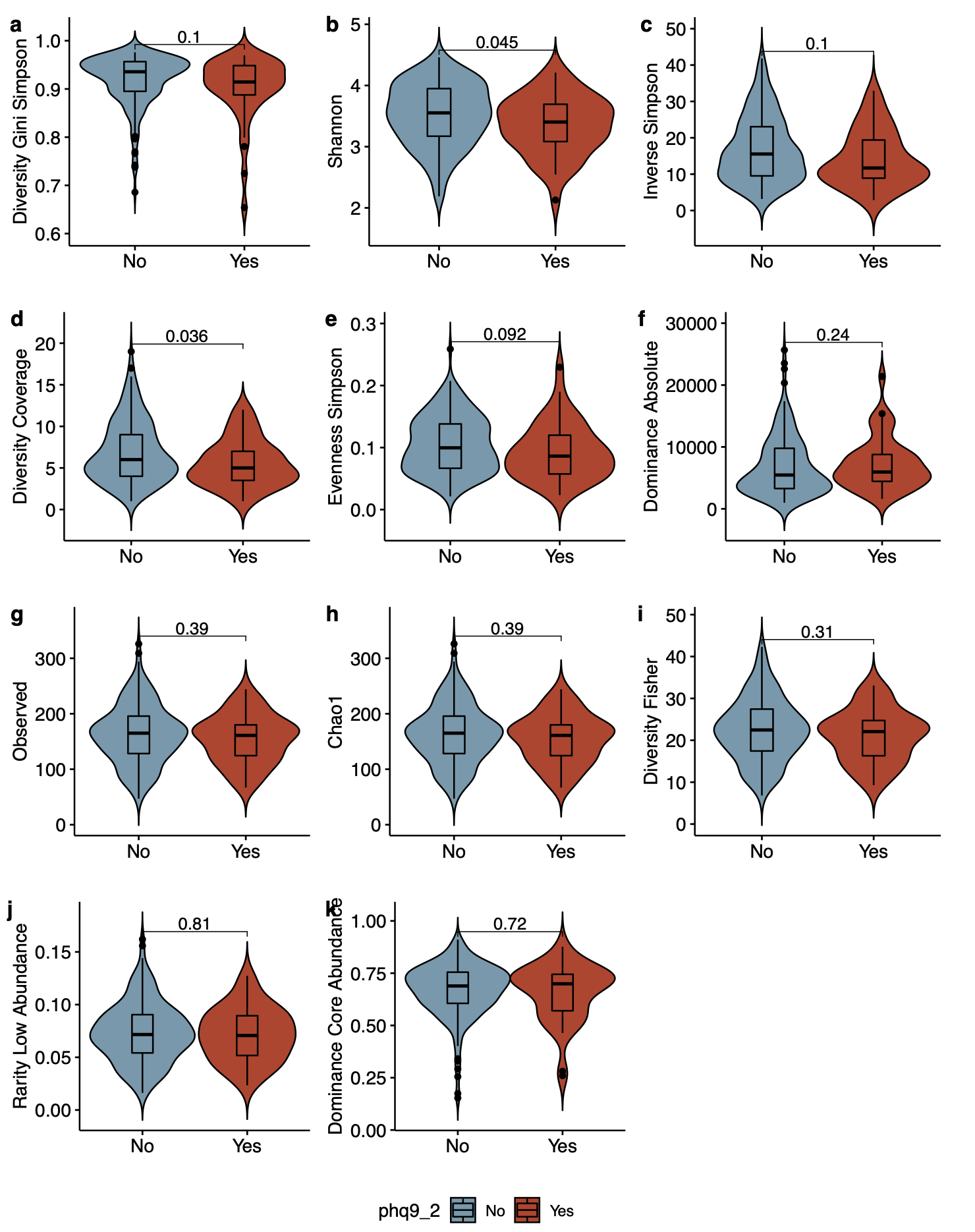


**Supplementary Fig. 1. Microbiome diversity differences in suicidal ideation (SI) endorsement concurrent with the presence of sleep issues.** Microbiome diversity indices in the N_sleep_ cohort, which includes only those endorsing sleep issues (non-zero score on question 3 of the PHQ-9) to control for the effect of sleep. The diversity measures that are indicated were compared across individuals with suicidal ideation (SI_sleep_= 43, “Yes”) and those without (NOSI_sleep_ = 186, “No”). Diversity measures were derived with the microbiome R package. Significant differences were calculated using a non-parametric Wilcoxon test in ggpubr

**
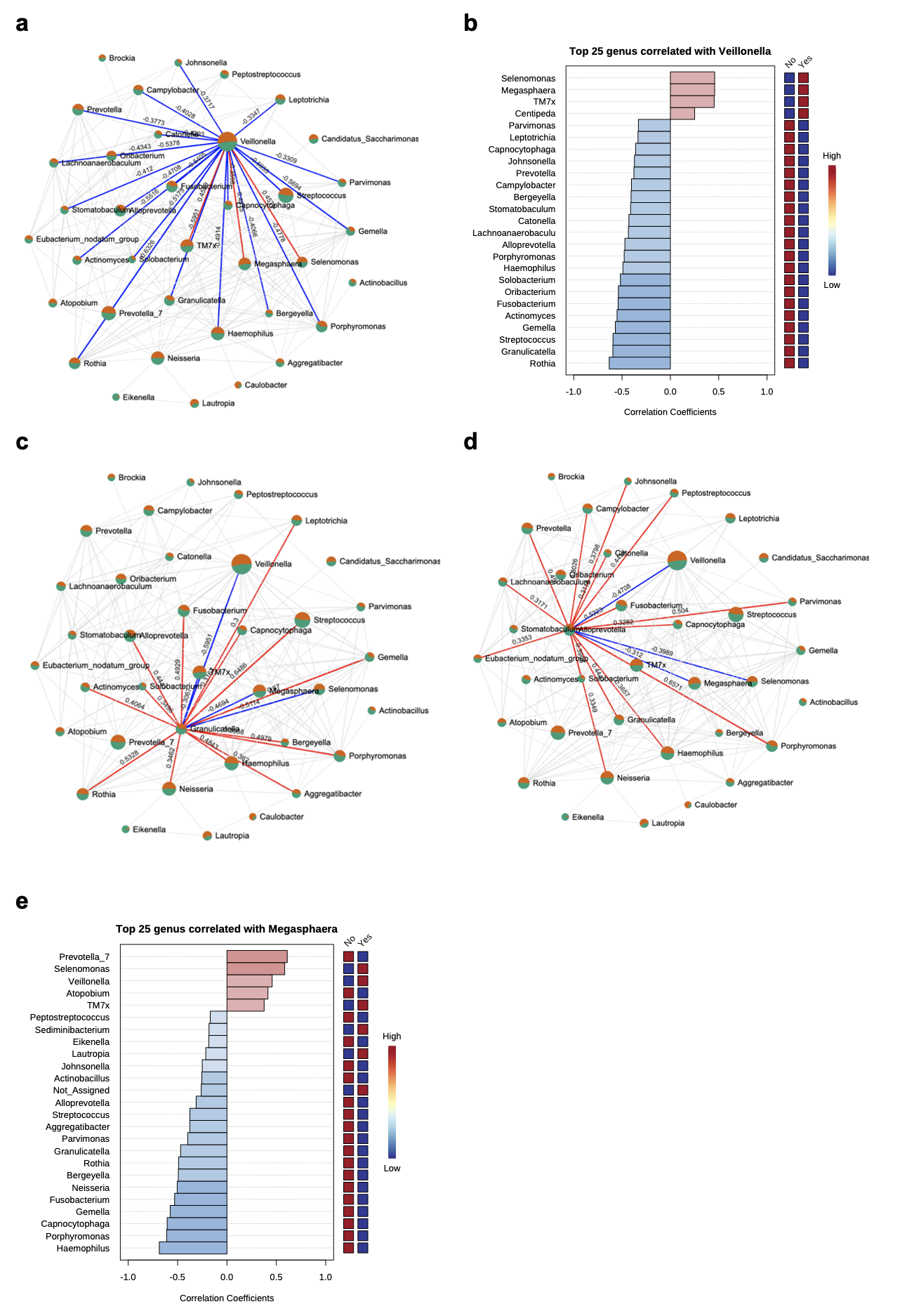
**

**Supplementary Fig. 2. Network correlation analysis of salivary genera after controlling for presence of sleep issues.** Bacteria in the saliva were assessed in the context of genera networks using correlation analysis while controlling for the presence of sleep issues. The analysis was restricted to only those 229 subjects who endorsed recent sleep issues on question 3 of the PHQ-9 (N_sleep_), to control for the effects of sleep on the microbial community. Spearman correlation coefficients are presented for statistically significant correlations with *Veillonella* (a,b), *Granulicatella* (c), *Alloprevotella* (d), and *Megasphaera* (e). For the network plots (a,c,d), correlations to the genus of interest are provided with a connection to the respective, correlated genus in either red (positive correlation) or blue (negative correlation). In b,e, the top 25 genera that correlated in either direction with the genus are presented, with the correlation coefficient indicated; on the right, genera are additionally labelled with a red or blue box, which indicates whether the genus is more or less abundant, respectively, when comparing those with SI and sleep issues, SI_sleep_ (yes) or those with sleep issues but no SI, NOSI_sleep_ (no). In (a,c,d), pie charts indicate the distribution of the genus with respect to those with SI (green) and those without SI (orange), with the size of the pie chart reflecting the relative observed abundance of the genus in the overall salivary community. Spearman correlations across all genera are provided in Supplementary Material.

**
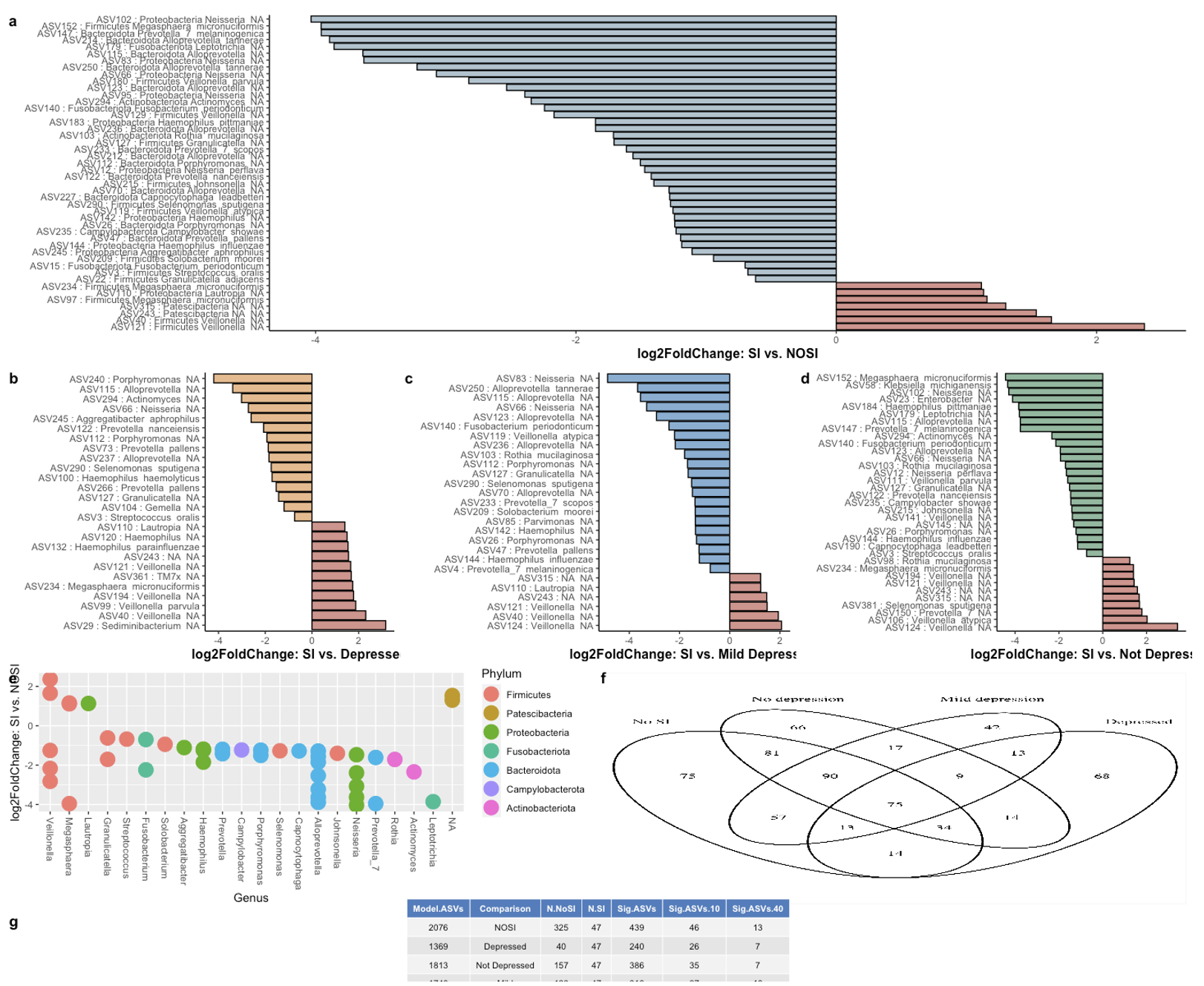
**

**Supplementary Fig. 3. Differentially abundant amplicon sequence variants (ASVs) from saliva in recent suicidal ideation (SI) and depression.** Using DESeq2, normalized base mean counts of the ASVs observed in the saliva community were compared in four methods: 1) the full cohort: those with SI compared to those with no SI, NOSI, and then selected cohorts, where those with SI were compared to 2) those with PHQ-9 scores above 10 (the published threshold for MDD), DEP, 3) those with PHQ-9 scores <5, NODEP, and 4) those with PHQ-9 scores between 5-9, MILD), separately. Significantly differentially abundant ASVs (FDR p-value ≤ 0.05) that have normalized base means ≥10 are presented in (a-d), comparing SI (N=47) to all individuals without SI, NOSI (a), and then to each of the following subgroups of NOSI separately: Depressed (b), Mild Depression (c), and Not Depressed (d) cohorts. Positive log2FoldChanges are indicative of higher expression in subjects with SI. Of the ASVs identified in this analysis, 75 were shared across the four models with any normalized base mean count and FDR p-value ≤ 0.05. The ASVs in (a) are presented with their taxonomy in (f) for the SI vs. NOSI comparison. Model summaries are presented in (g), including the number of ASVs entered in the model after independent filtering, the comparison groups and their N, and the number of significant ASVs at any base mean count, ≥10, and ≥40. The full DESeq2 model statistics for all significantly differentially expressed ASVs are presented in Supplemental Material.

**Supplementary Table 8. Differentially abundant taxa in suicidal ideation (SI) in a restricted cohort after controlling for sleep issues: SI_sleep_ compared to NOSI_sleep_.** DESeq2 results of differential abundance analysis on N_sleep_ (N=229) comparing bacterial communities observed in SI (SI_sleep_= 43) and NOSI (NOSI_sleep_ = 186) among those with sleep issues (non-zero response on PHQ-9:3). 75 of 227 genera and 219 of 560 species were fitted in the model after independent filtering. Negative log2 fold changes values indicate bacteria higher in controls NOSI_sleep_, positive values in SI_sleep_.

| Phylum | Genus | Species | Higher in | baseMean | log2FoldChange | pvalue | padj |
| --- | --- | --- | --- | --- | --- | --- | --- |
| Firmicutes | *Granulicatella* | --- | NOSI_sleep_ | 241.857 | -0.903 | 4.80E-05 | 0.00913 |
| Proteobacteria | *Klebsiella* | *michiganensis* | NOSI_sleep_ | 12.371 | -24.863 | 4.56E-18 | 6.66E-16 |
| Proteobacteria | *Klebsiella* | *aerogenes* | NOSI_sleep_ | 19.040 | -25.273 | 9.29E-12 | 6.78E-10 |
| Bacteroidetes | *Prevotella* | *melaninogenica* | NOSI_sleep_ | 1800.632 | -1.202 | 3.80E-05 | 0.00185 |
| Firmicutes | *Granulicatella* | *adiacens* | NOSI_sleep_ | 346.459 | -1.169 | 0.00018 | 0.00531 |
| Firmicutes | Gemella | NA | NOSI_sleep_ | 148.003 | -1.245 | 0.00018 | 0.00531 |
| Bacteroidetes | Porphyromonas | NA | NOSI_sleep_ | 549.054 | -1.113 | 0.00067 | 0.01641 |
| Bacteroidetes | *Capnocytophaga* | *leadbetteri* | NOSI_sleep_ | 40.188 | -1.433 | 0.00092 | 0.01925 |
| Firmicutes | *Megasphaera* | *micronuciformis* | SI_sleep_ | 1564.587 | 1.203 | 0.00163 | 0.02637 |
| Bacteroidetes | *Prevotella* | *intermedia* | NOSI_sleep_ | 14.129 | -3.205 | 0.00157 | 0.02637 |

**Supplementary Table 9. Human Leukocyte Antigen (HLA) Class II associations with suicidal ideation (SI).** Mean precent relative abundances of selected species in subjects with the indicated HLA class II haplotype. The indicated genotype codes 0, 1, and 2 correspond to absence of the allele, heterozygosity of the allele, and homozygosity of the allele, respectively, at each haplotype. The DQ2.2 and DQ2.5 haplotypes are defined by the presence of DQB1*02 and DQA1*02 or DQA1*05, respectively. DQ8 is defined by the presence of both DQA1*03 and DQB1*03. DR4 is synonymous with DRB1*04.

|  | DPA1*01 | | | DRB1*03 | | | DRB1*15 | | |
| --- | --- | --- | --- | --- | --- | --- | --- | --- | --- |
|  | 0 alleles | 1 allele | 2 alleles | 0 alleles | 1 allele | 2 alleles | 0 alleles | 1 allele | 2 alleles |
|  | Mean | Mean | Mean | Mean | Mean | Mean | Mean | Mean | Mean |
| *Prevotella melaninogenica* | 2.8865 | 4.1221 | 4.1558 | 3.8927 | 4.0253 | 8.7306 | 3.9912 | 4.0954 | 3.9621 |
| *Neisseria mucosa* | 0.5478 | 0.7238 | 0.5600 | 0.6193 | 0.6160 | 0.9950 | 0.6191 | 0.4541 | 1.8217 |
| *Rothia mucilaginosa* | 0.9330 | 0.9458 | 1.5360 | 1.2338 | 1.3621 | 1.4552 | 1.1780 | 1.4327 | 1.7492 |
|  |  |  |  |  |  |  |  |  |  |
|  | DQ2.2 and DQ2.5 | | DQ8 (DQA1*03 and DQB1*03) | | DR4 and DQ8 | |  |  |  |
|  | not DQ2.2 and DQ2.5 | DQ2.2 and DQ2.5 | Not DQ8 | DQ8 | Not DR4 and DQ8 | DR4 and DQ8 |  |  |  |
|  | Mean | Mean | Mean | Mean | Mean | Mean |  |  |  |
| *Megasphaera micronuciformis* | 2.8516 | 4.6007 | 3.1301 | 2.4830 | 3.0714 | 2.6308 |  |  |  |
| *Enterobacter kobei* | 0.2905 | 0.0000 | 0.0304 | 0.9615 | 0.0293 | 1.3951 |  |  |  |
| *Klebsiella aerogenes* | 0.9179 | 0.0000 | 0.0813 | 3.0798 | 0.0784 | 3.3056 |  |  |  |
| *Haemophilus haemolyticus* | 0.1760 | 0.4365 | 0.1795 | 0.2295 | 0.1785 | 0.2396 |  |  |  |
| *Haemophilus pittmaniae* | 0.1226 | 0.4200 | 0.1561 | 0.0994 | 0.1557 | 0.0950 |  |  |  |
| *Veillonella rogosae* | 0.1350 | 0.9943 | 0.2347 | 0.0597 | 0.2298 | 0.0660 |  |  |  |
